# Systematic prediction of degrons and E3 ubiquitin ligase binding via deep learning

**DOI:** 10.1186/s12915-022-01364-6

**Published:** 2022-07-14

**Authors:** Chao Hou, Yuxuan Li, Mengyao Wang, Hong Wu, Tingting Li

**Affiliations:** 1 Department of Biomedical Informatics, School of Basic Medical Sciences, Peking University Health Science Center, Beijing, 100191 China; 2 Key Laboratory for Neuroscience, Ministry of Education/National Health Commission of China, Peking University, Beijing, 100191 China; 3 The MOE Key Laboratory of Cell Proliferation and Differentiation, School of Life Sciences, Peking University, Beijing, 100871 China; 4 Peking-Tsinghua Center for Life Sciences, Beijing, China; 5 Institute for Cancer Research, Shenzhen Bay Laboratory, Shenzhen, China

**Keywords:** Degron, E3 Ubiquitin ligase, Protein degradation, Deep learning, Cancer driver mutation

## Abstract

**Background:**

Degrons are short linear motifs, bound by E3 ubiquitin ligase to target protein substrates to be degraded by the ubiquitin-proteasome system. Mutations leading to deregulation of degron functionality disrupt control of protein abundance due to mistargeting of proteins destined for degradation and often result in pathologies. Targeting degrons by small molecules also emerges as an exciting drug design strategy to upregulate the expression of specific proteins. Despite their essential function and disease targetability, reliable identification of degrons remains a conundrum. Here, we developed a deep learning-based model named Degpred that predicts general degrons directly from protein sequences.

**Results:**

We showed that the BERT-based model performed well in predicting degrons singly from protein sequences. Then, we used the deep learning model Degpred to predict degrons proteome-widely. Degpred successfully captured typical degron-related sequence properties and predicted degrons beyond those from motif-based methods which use a handful of E3 motifs to match possible degrons. Furthermore, we calculated E3 motifs using predicted degrons on the substrates in our collected E3-substrate interaction dataset and constructed a regulatory network of protein degradation by assigning predicted degrons to specific E3s with calculated motifs. Critically, we experimentally verified that a predicted SPOP binding degron on CBX6 prompts CBX6 degradation and mediates the interaction with SPOP. We also showed that the protein degradation regulatory system is important in tumorigenesis by surveying degron-related mutations in TCGA.

**Conclusions:**

Degpred provides an efficient tool to proteome-wide prediction of degrons and binding E3s singly from protein sequences. Degpred successfully captures typical degron-related sequence properties and predicts degrons beyond those from previously used motif-based methods, thus greatly expanding the degron landscape, which should advance the understanding of protein degradation, and allow exploration of uncharacterized alterations of proteins in diseases. To make it easier for readers to access collected and predicted datasets, we integrated these data into the website http://degron.phasep.pro/.

**Supplementary Information:**

The online version contains supplementary material available at 10.1186/s12915-022-01364-6.

## Background

The ubiquitin-proteasome system (UPS) dynamically regulates protein turnover in cell differentiation, cell cycle, and signaling pathways [1, 2], with over 80% of intracellular proteins being degraded via UPS [3]. During the degradation process, ubiquitin (Ub) is covalently attached to lysine (K) on the substrate, which is catalyzed by E1 ubiquitin-activating enzymes, E2 ubiquitin-conjugating enzymes, and E3 ubiquitin ligases [4]. Subsequently, the ubiquitinated substrate is transferred to and degraded by the 26S proteasome [5]. The human genome encodes two E1s, 41 E2s, and more than 600 E3s [6]. E3s bind their substrates directly via E3 binding sites present on the surface of substrates. These binding sites are called degrons [7]. The interaction between E3 and degron determines the specificity of the degradation process.

Degrons are preferentially located in disordered regions and are molecular recognition features (MoRFs) that undergo disorder-to-order transition upon binding to E3s [8]. Degrons are typically regulated by post-translational modifications (PTMs), which control the interaction with E3s in response to environmental and cellular cues [9]. Degrons mediate the ubiquitination of substrates, and the resulting Ub-sites are usually located within 20 amino acids (AAs) distant from the degron [7]. A fundamental property of degrons is their transferability: in most cases, transplantation of a degron to a protein accelerates the degradation of a protein [10]. In contrast, dysfunction of degrons disturbs control of protein degradation and causes abnormally accumulating proteins, thus further contributing to pathological progression [1, 11]. This situation particularly applies to cancer, a disease that involves the enhanced expression of oncogenes. Recently, researchers explored the targetability of degrons by designing small molecules for a degron on tumor suppressor p53. Two resulting small molecules upregulated p53 expression and restored p53 function, which provide an opportunity to inhibit cancer cell growth [12]. Thus, identifying degrons on the substrates should greatly assist in investigating the pathogenesis of related diseases and provide potential therapeutic targets.

Both low-throughput and high-throughput experimental approaches have been employed to identify degrons. Low-throughput identification of degrons usually require deletion or mutation of specific sites on proteins coupled with half-life experiments or co-immunoprecipitation with specific E3s [13]. However, many degrons are exposed to E3s only when proteins fail to fold correctly [14] and thus fail to interact with E3s or prompt degradation on well-folded proteins. In addition, the interactions between E3s and substrates are temporary [11, 15]. Thus, low-throughput identification of degrons faces many challenges, and only a limited number of degrons have so far been identified [16]. Recently, high-throughput methods were designed to identify degrons as well. These methods were based on the transferability of degrons [14] and considered the peptides that promote the degradation of a reporter protein as degrons [10, 17]. For example, Elledge and colleagues developed a bimodal fluorescent expression cassette termed Global Protein Stability (GPS) to discover N-end and C-end degrons [10, 17]. However, this method did not identify internal degrons, and it is almost impossible to screen all peptides in the human proteome. Notably, flexible segments facilitating access to the catalytic core of proteasome and peptides prone to be ubiquitinated can also promote the degradation of the reporter protein [7, 18, 19]. As a consequence, the destabilizing peptides identified via high-throughput methods may represent not only degrons but also other functional peptides that accelerate protein degradation [7, 18, 19]. Given the complexity and difficulty of experimental identification of degrons, an efficient predictor is urgently required to facilitate degron discovery.

However, only a few bioinformatic tools have been developed to predict possible degrons. Motif matching is widely used in predicting degrons, and 25 degron motifs from the ELM motif database are commonly used [16]. While motif matching excels in predicting possible degrons fast from local sequence patterns, it fails to consider other critical features such as structure or solvent accessibility of specific sites, which leads to high false-positive rate of motif matches. To reduce the false-positive rate of motif matching, Martínez-Jiménez et al. scored all internal motif matches in human proteome using a random forest classifier (hereafter Motif_RF) with 11 biochemical features, including flanking phosphorylation sites, disordered regions, MoRFs, solvent accessibility, conservation, secondary structure, and flanking ubiquitinated Ks [20]. Motif_RF identified over 20,000 likely new degrons in different protein isoforms. Nevertheless, Motif_RF failed to predict terminal degrons and could not be applied to proteins without available PTM data. Besides, only less than 30 E3 motifs are available for motif-based methods, which cannot even cover degrons for 5% of more than 600 E3s, precluding us from identifying degrons bound by other E3s. Tokheim et al. trained a deep learning model deepDegron on high-throughput GPS data to predict N-end and C-end degrons [11]. deepDegron can only predict terminal degrons, and its training set derived from GPS experiment not only includes degrons. Thus, the predicted results of deepDegron represent multiple destabilizing peptides. Overall, exiting degron predictors are limited by either a high false positive rate or a limited application range. A more general model is needed to give a broader and unbiased prediction of degrons.

Besides identifying degrons, identifying E3-substrate interactions (ESIs) is also an area of intense study. Experiments like co-immunoprecipitation, two-hybrid screening, and mass spectrometry are commonly used to discover new ESIs [15]. Recently, machine learning-based ESI predictors were developed as well. Wang et al. developed Ubibrowser 2.0 [21] to predict ESIs using the enriched domain, GO term pair, protein-protein interaction, and inferred E3 recognition consensus motif; Chen et al. [22] built a machine learning model (hereafter ChenESI) to predict ESIs from proteomics data, transcriptomics data, protein-protein interaction, and pathway-based associations. However, both experimental methods and prediction methods lack binding degron information. Here, we solved this restriction by combining our degron predictor and E3 motifs.

In this work, we predict degrons using a BERT-based deep learning approach. Our newly designed model Degpred successfully captures degron-related sequence properties and considerably expands the degron landscape. By assigning predicted degrons to E3s using our generated E3 motifs, we predicted ESIs with binding degron information. We also investigated the significance of degrons and binding E3s in protein turnover and tumorigenesis.

## Results

### The BERT-based model predicts degrons of new sequence patterns

**Fig. 1:**
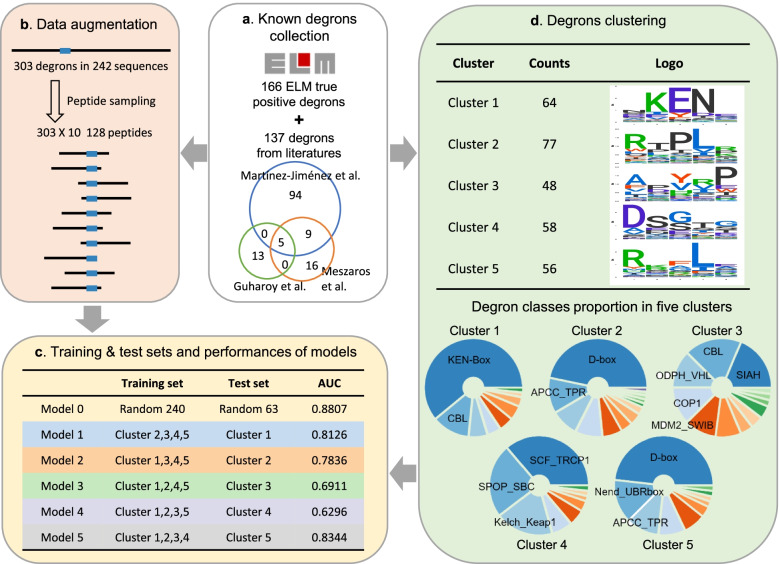
Degron collection, processing, and model performance. **a** Degrons were collected from the true positive degrons in the ELM database and three previous studies. Venn diagram showing the overlap of collected degrons from three previous studies. **b** Data augmentation: sampling 128AA-peptides around degrons from original protein sequences. The blue rectangles represent degrons, and the black lines represent peptide sequences. **c** Training sets, test sets, and performances of models, models were evaluated with the area under the receiver operating characteristic curve (AUC). **d** Degron clustering and composition of five clusters, Seqlogos in the upper panel show the sequence patterns of alignment cores for five clusters in Gibbscluster, classes accounting for at least 10 percent in each cluster are labeled in pie graphs

To train and evaluate models, we collected known degrons from ELM [16] database and three previous studies [1, 7, 20] (Fig. 1a). For the same degrons present on different isoforms of one gene, only main isoforms in UniProt [23] were reserved. In total, 303 degrons typically spanned 5-10 AAs were obtained (Additional file 1: Fig. S1a, Additional file 2: Table S1).

Previous predictors predict degrons by integrating protein features like flanking phosphorylation sites, intrinsically disordered regions, MoRFs, solvent accessibility, and flanking ubiquitinated Ks [20, 24]. However, these models cannot be applied to proteins without PTM data, and annotating proteins with these features is time-consuming. Thus, we turned to using BERT-based deep learning models, which have been shown to successfully represent fundamental and advanced properties of proteins, including secondary structure, target binding sites, contact, and PTMs [25, 26]. We built a BERT-based model to predict degrons that consists of a pre-trained TAPE BERT-encoding model [27], two bidirectional long short-term memory layers, and two fully connected layers. The architecture took a protein sequence as input and outputted scores for all AAs on the protein (Additional file 1: Fig. S1b, see Methods for details).

To explore the feasibility of using the BERT-based model to predict degrons, we compared the ability of our model with Motif_RF and a general MoRF predictor MoRFchibi [8] to classify degrons from motif matches. In the training and test stages, our collected degrons were labeled as 1, while randomly selected motif matches from proteins without known degrons were labeled as 0. We averaged predicted scores of AAs in degrons or negative motif matches to represent the score of the BERT-based model. The BERT-based model achieved comparable performance with Motif_RF under fivefold cross-validation, and both methods significantly outperformed MoRFchibi (Additional file 1: Fig. S1c, see Methods for detail). This result indicated that the BERT-based model provides an alternative to protein feature integrating predictors. The advantage of the BERT-based model is that it only needs protein sequence as input and has broader scope of application.

As degrons derived from motifs only represent a small proportion of all degrons bound by more than 600 E3s, we next used our model to score all AAs in sequences rather than only AAs in motif matches of a limited number of motifs. To provide more inputs for the training of the deep learning model, we augmented known degrons by sampling peptides from original proteins. We randomly sampled ten peptides of 128 AAs containing the degron from the original protein for each degron, and generated 3030 128AA-peptides containing known degrons in total (Fig. 1b). As transplantation of a degron confers instability on other proteins [10], we reasoned that degrons on 128AA-peptides can mediate the degradation of 128AA-peptides as well. Thus, we used these 128AA-peptides to train our model. In the training and test stages, AAs in known degrons were labeled as 1, while AAs in the other regions were labeled as 0 (see Methods for detail). We first trained a model (model 0) on 128AA-peptides from 240 randomly selected degrons and tested it on the other 63 degrons. As shown in Fig. 1c, model 0 attained an AUC of 0.8807. This result suggested that we can use the BERT-based model to predict degrons from protein sequences rather than only from motif matches.

Next, we explored whether the BERT-based model can predict degrons bound by E3s not present in our dataset. If the model trained on known degrons bound by a set of E3s can predict that of other E3s, we can infer that our model can discover degrons of new classes. Ideally, degrons used for training and test should be dissimilar in sequence. As it is hard to measure the similarity of degrons bound by different E3s, we grouped 303 known degrons into five clusters using sequence alignment [28]. As shown in Fig. 1d, clusters 1, 2, 4, and 5 possessed dominant classes accounting for about 50 percent, while cluster 3 lacked dominant class and acted as a trash bin during clustering. Next, we built five models, trained each model on degrons from four clusters, and tested each model on degrons of the remaining cluster (Additional file 1: Fig. S1d,e,f). As shown in Fig. 1c, models 1, 2, and 5 performed well in predicting degrons dissimilar with training degrons. Given the diversity of degrons in cluster 3, the performance of model 3 was also satisfactory. The dominant class in cluster 4 is phosphorylation-dependent degrons SCF_TRCP1 [29] (Fig. 1d); as the training degrons of model 4 are mostly modification-independent, model 4 might ignore PTM-related information in the BERT-encoding matrix and performed relatively poorly in predicting phospho-degrons. Overall, these results suggested that even though degrons in different clusters have little sequence homology, they share features beyond the primary sequence that can be captured by the BERT-based model.

To evaluate the importance of information in the BERT-encoding matrix in predicting degrons, we compared the BERT-based model with a new predictor possessing similar architecture and number of trainable parameters, except that it took one-hot encoding as input (Additional file 1: Fig. S1g). We trained and tested the one-hot model using the same strategy as the BERT-based model and found that the BERT-based model significantly outperformed the one-hot model in predicting degron in five clusters (Additional file 1: Fig. S1d,e,h). This result suggested that the rich information encoded in the TAPE BERT-encoding matrix helps our model discover novel degrons dissimilar to training degrons.

In summary, these findings suggested that the BERT-based model can be used as an alternative to feature integrating degron predictors and has wilder scope of application. In addition, our model can predict degrons of new sequence patterns with satisfy performance; thus, it can be used to discover new degrons proteome-widely.

### Degpred expands the degron landscape and assists in identifying degrons from motif matches

**Fig. 2:**
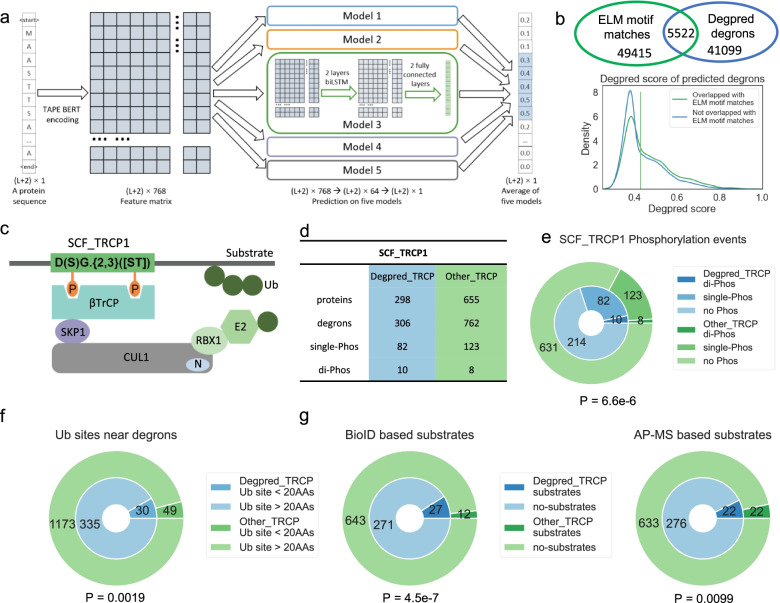
Degpred expands the degron landscape and assists in identifying degrons from motif matches. **a** A schematic diagram to illustrate the process of Degpred in predicting degrons, the dimensions of matrixes and vectors in the prediction process are labeled below. **b** Upper penal: Venn diagram showing the counts and overlap of Degpred degrons and ELM motif matches in the human proteome. Lower penal: distribution of Degpred scores of predicted degrons overlapped and not overlapped with ELM motif matches. The green vertical line represents the median score of overlapped degrons. **c** A schematic representation of how βTrCP binds substrates, di-phosphorylation on the motif match is necessary for the binding. Mammal genomes encode βTrCP1 and βTrCP2 that recognize the same degron motif [29], we did not distinguish them in our analysis. **d** Statistics of βTrCP motif matches overlapped with Degpred degrons (Degpred_TRCP, blue) or not overlapped with Degpred degrons (Other_TRCP, green). **e** Counts of phosphorylation sites occurring on Degpred_TRCP and Other_TRCP. *P*-value was calculated using the chi-square test. **f** Counts of Ub-sites occurring near Degpred_TRCP and Other_TRCP. *P*-value was calculated using Fischer's exact test. **g** Comparison of Degpred_TRCP and Other_TRCP containing proteins in BioID and AP-MS based high-throughput βTrCP substrates. *P*-values were calculated using Fischer’s exact test

Models 1–5 trained on degrons with different sequence patterns represent different aspects of degron properties. Thus, we assembled models 1–5 to build Degpred to take full advantage of known degrons and provide more comprehensive predictions (Fig. 2a). Degpred averages outputs from five models to score all AAs of the input protein. Taking 0.3 as the cut-off, Degpred attained a false discovery rate (FDR) of 0.512 (Additional file 1: Fig. S2a) and predicted 46,621 degrons present in the human proteome (UniProt [23] human reviewed proteins) (Additional file 2: Table S1).

To provide an overview of degrons predicted by Degpred, we first compared Degpred degrons with about 55,000 ELM motif matches in the human proteome and found that only 5522 Degpred degrons overlap with ELM motif matches (Fig. 2b). We further analyzed the averaged Degpred score of degrons that match ELM motifs and degrons that do not match ELM motifs. As shown in Fig. 2b, more than 41% of not overlapped degrons possess Degpred scores higher than the median score of overlapped degrons. Even though most training degrons were initially identified through ELM motifs, over 88% of Degpred degrons were beyond those discovered using motifs. These results suggested that Degpred expands the degron landscape. Next, we investigated the relationship between terminus located Degpred degrons and N-end and C-end destabilizing peptides in high-throughput GPS experiments [10, 17], which also constitute the training set of deepDegron [11]. Unexpectedly, we found that both Degpred degrons and known degrons tend to act as stabilizing peptides in GPS experiments (Additional file 1: Fig. S2b). This discordance might be because destabilizing peptides in the GPS experiment are a mixture of multiple functional peptides not limited to degrons [7, 18, 19]. Further investigations are needed to explore the underlying mechanism of destabilizing peptides in the high-throughput experiment.

Another major disadvantage of motif matching constitutes its high false-positive rate due to only considering local sequence patterns. We investigated whether Degpred can screen real degrons from motif matches by testing Degpred on the motif matches of extensively studied E3 βTrCP. The degron of βTrCP requires a special sequence patterns and di-phosphorylation to be recognized [29, 30] (Fig. 2c). The motif of βTrCP matches 1068 segments on 953 proteins in the human proteome, and 306 matches on 298 proteins overlap with Degpred degrons (Fig. 2d). To compare the possibility of motif matches with and without Degpred signal functioning as degrons, we first surveyed phosphorylation sites in the database PhosphoSitePlus [31] and Ub-sites in the database dbPTM [32]. Because real degrons bound by βTrCP possess two phosphorylation sites and are rich in Ub-sites located within 20 AAs [7]. As shown in Fig. 2e, a higher proportion of Degpred-screened matches were phosphorylated compared to the other matches, both single-phosphorylation and di-phosphorylation. Moreover, we found that Ub-sites were significantly enriched within 20 AAs of Degpred-screened matches compared to the other matches (Fig. 2f). Next, we analyzed potential βTrCP substrates identified by proximity-dependent biotin labeling (BioID) [30] and affinity purification mass spectrometry (AP-MS) [29]. As shown in Fig. 2g, proteins with Degpred-screened matches were identified at higher rates in both experiments compared to proteins with the other matches. These results suggested that Degpred helps identify real degrons from motif matches.

Overall, our deep learning degron predictor Degpred identifies novel degrons with new sequence patterns and helps reduce the false-positive rate of motif matches.

### Degpred degrons exhibit typical degron properties and are rich in ubiquitination sites nearby

**Fig. 3:**
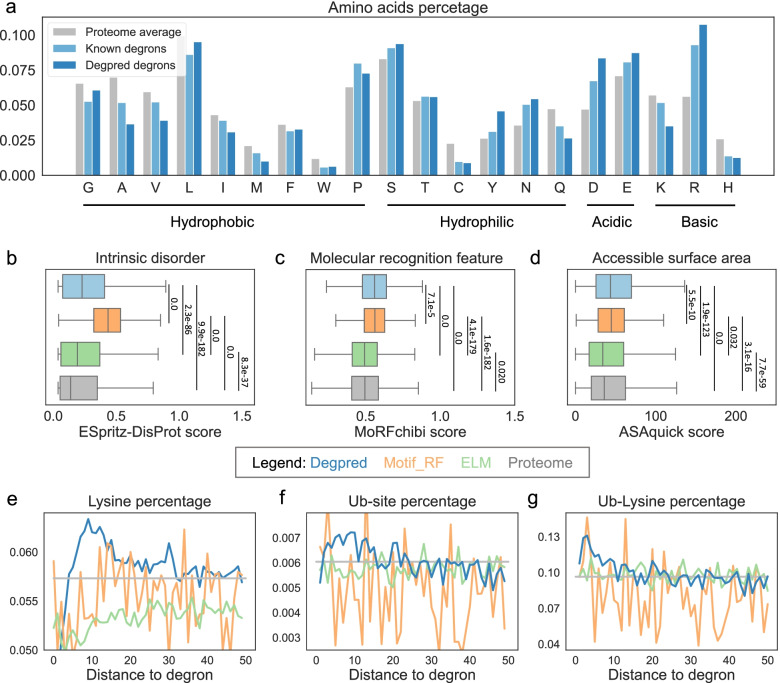
Properties of Degpred degrons and comparison with motif-based methods. **a** The AA composition of known degrons, Degpred degrons, and the human proteome, AAs are grouped based on their properties. **b–d** Distributions of predictions of intrinsic disorder, molecular recognition feature, and accessible surface area of predicted degrons in Degpred (blue), Motif_RF (orange), ELM motif matching (green), and random peptides from human proteome (gray, see Methods for detail). The intrinsic disorder was predicted with ESpritz-DisProt; the molecular recognition feature was predicted with MoRFchibi; the accessible surface area was predicted with ASAquick. *P*-values were calculated using two sides *T*-test. **e–g** Percentages of lysine, Ub-site, and Ub-lysine in the flanking 50 AAs of predicted degrons in Degpred (blue), Motif_RF (orange), and ELM motif matching (green), averages of the human proteome are displayed in gray. Both sides of predicted degrons were considered

To explore the properties of predicted degrons, we first analyzed the AA composition of Degpred degrons and known degrons. As shown in Fig. 3a, the AA composition of Degpred degrons resembles that of known degrons. Proline (P), glutamic acid (E), serine (S), and tyrosine (T) which were reported to be enriched in degradation signals [33] were all enriched in Degpred degrons; S, T, and tyrosine (Y) which can be phosphorylated were enriched in Degpred degrons as well. Further analysis showed that not only phosphorylation sites, but also N-linked Glycosylation and Methylation sites were enriched in Degpred degrons (Additional file 1: Fig. S2c). These results indicated that Degpred successfully learns the correct AA preference of known degrons, and suggested that some PTMs might act as degron regulators and cross-talk with ubiquitination.

Furthermore, we compared the properties of predicted degrons of Degpred, ELM motif matching and Motif_RF [20]. As Motif_RF utilized 11 features including intrinsically disordered regions, MoRFs, solvent accessibility, and flanking ubiquitinated Ks to predict possible degrons, we first compared these sequence properties of predicted degrons of three methods. As expected, Motif_RF predicted degrons scored higher in the predictions of intrinsically disordered regions, MoRFs, and solvent accessibility [8, 34, 35] than ELM motif matches and random peptides from the human proteome (Fig. 3b–d). Surprisingly, Degpred degrons also scored higher in these predictions (Fig. 3b–d), which indicates that Degpred captures correct sequence features of degrons. Next, we surveyed Ks and Ub-sites [32] around predicted degrons of three methods. As shown in Fig. 3e, Ks were enriched around Degpred degrons, which provides a suitable environment for E3s to ubiquitinate substrates after binding to degrons. In addition, we found that both Ub-sites and ubiquitinated Ks were enriched around Degpred degrons as well (Fig. 3f, g). In comparison, Ks, Ub-sites, and ubiquitinated Ks were randomly distributed around ELM and Motif_RF predicted degrons. These results indicated that Degpred degrons might mediate ubiquitination of flanking Ks.

In summary, Degpred degrons exhibit typical degron properties and might promote ubiquitination of nearby Ks, supporting the assumption that Degpred degrons constitute the binding sites of E3s.

### Predicting binding E3s of degrons using calculated motifs

**Fig. 4:**
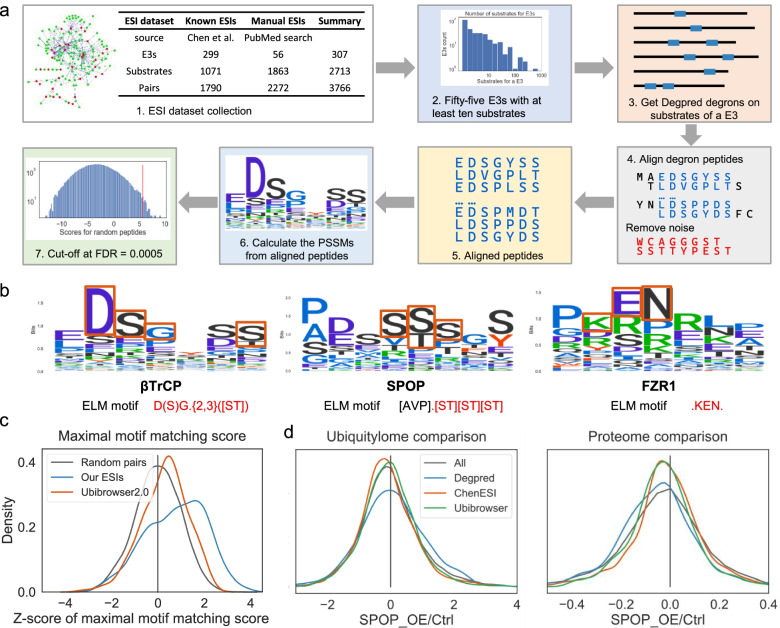
E3 motifs calculation and evaluation. **a** Workflow of motifs calculation. 1. Summary of the ESI dataset collected from PubMed and related works. 2. Fifty-five E3s possessing at least ten substrates in the collected ESI dataset were selected for motif calculation. 3. Predicting Degpred degrons on substrates in the ESI dataset for each E3. 4. Degron alignment and outlier removal with GibbsCluster for each E3. 5. Aligned regions present on degrons were considered to represent the binding sites for each E3. 6. Motif calculation using aligned peptides for each E3. 7. Cut-off calculation, degrons with motif matching scores larger than the cut-off were predicted as binding degrons of the E3. **b** Comparison of our generated motifs of βTrCP, SPOP, and FZR1 with their experimentally verified motifs in the ELM database. ELM motifs are represented as regular expressions, and consistent regions are highlighted in red. **c** Distribution of normalized maximal motif matching scores for our ESIs, the ESIs collected in Ubibrowser2.0 and not in our ESIs, and random pairs. Kernel density estimate plot showing the distribution of scores. The maximal motif matching scores were normalized to *Z*-scores for each E3 respectively. **d** Comparison of Ub-site abundance change and protein abundance change of predicted SPOP substrates in three methods after SPOP overexpression. Abundances of replicate 1 in original paper [38] were compared, and the results of replicate 2 were identical (data not shown). ChenESI predicted 1195 SPOP substrates and 867 of them were detected in the SPOP overexpression MS; Ubibrowser2.0 predicted 892 SPOP substrates and 622 of them were detected in the MS; we selected the top 2000 predicted SPOP substrates and 669 of them were detected in the MS. Kernel density estimate plots showing the distribution of the ratios after and before SPOP overexpression

After predicting degrons, we set out to predict the regulatory E3s for Degpred degrons. The most straightforward method is to match degrons with E3 motifs as used in motif-based methods, but only a small number of experimentally identified E3 motifs were available. Here, we computationally generated E3 motifs using Degpred degrons on substrates in our collected E3-substrate interactions (ESIs) dataset (Fig. 4a, Additional file 1: Fig. S3a, Additional file 3: Table S2, see Methods for detail). We chose 55 E3s with at least ten substrates in the ESI dataset and calculated their motifs respectively. For each E3, we used GibbsCluster [28] to align Degpred degrons on its substrates and drop dissimilar outliers, which might be the binding sites of other E3s. Subsequently, we generated motifs from the aligned Degpred degrons for each E3 (Fig. 4a, see Methods for detail). As shown in Fig. 4b, the calculated motifs for βTrCP, SPOP, and FZR1 resemble their experimentally identified motifs [16]. In addition, we generated motifs for four HECT E3s (WWP1, WWP2, SMURF2, NEDD4L) which recognize proline-rich motifs through the WW domain [36, 37]. Four generated HECT E3 motifs were rich in proline (Additional file 1: Fig. S3b). These results indicated that our procedure to generate motifs is reliable.

To evaluate the ability of our generated motifs to predict ESIs, we defined a score to measure the binding possibility of an E3 and a substrate: we scored all Degpred degrons of the substrate with the E3 motif and selected the maximal motif matching score to represent the binding possibility. As shown in Fig. 4c, our collected ESIs possessed significantly higher scores than random pairs. In addition, the manually collected ESIs of Ubibrowser2.0 [21] not in our dataset also had higher scores. This finding indicated that our generated motifs could discover new ESIs. Furthermore, we compared generated motifs and ChenESI [22] on manually collected ESIs of Ubibrowser2.0. We found that generated motifs and ChenESI predicted similar number of substrates for SPOP and FZR1 (Additional file 1: Fig. S3d). Next, we compared generated motifs, Ubibrowser2.0 and ChenESI on ubiquitylome and proteome data measured after SPOP overexpression [38]. We found that SPOP substrates from the generated motif showed increased ubiquitination levels and reduced protein levels after SPOP overexpression (Fig. 4d). In contrast, the substrates of ChenESI and Ubibrowser2.0 showed no significant change. Thus, these results suggested that our generated motifs can be used to predict ESIs. More importantly, our generated motifs provide information of binding degrons which is absent in Ubibrowser2.0 and ChenESI.

Finally, we set out to construct a protein degradation regulatory network using Degpred degrons and generated motifs. We calculated cut-offs for motifs (Fig. 4a) and used the cut-offs to estimate whether an E3 will bind a predicted degron (see Methods for detail). To assess the ability of 55 generated motifs to discover real ESIs, we predict our collected ESIs using 55 motifs. We found that 71% (39/55) of motifs can predict at least 40% of collected substrates (Additional file 1: Fig. S3c, Additional file 4: Table S3). We selected these 39 motifs to construct a protein degradation regulatory network, which consists of 25695 ESIs between 39 E3s and 8754 substrates (Additional file 1: Fig. S3e, Additional file 4: Table S3).

In summary, we generated E3 motifs using Degpred and our collected ESI dataset. These motifs expanded known E3 motifs in the ELM database and enabled us to predict new ESIs with binding site information.

### E3-degron interactions affect half-lives of substrates

**Fig. 5:**
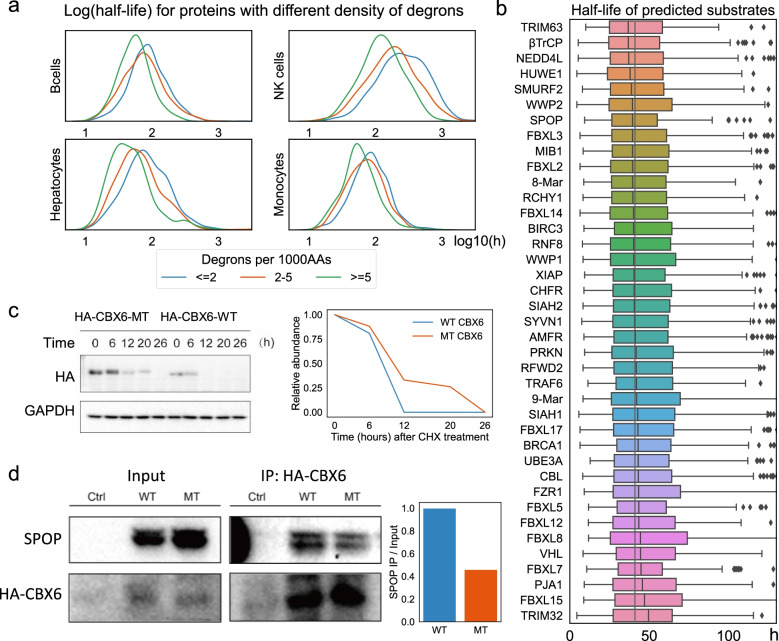
E3-degron interactions impact half-lives of substrates. **a** Distribution of half-lives for proteins with different densities of degrons in four non-dividing cell types. The half-lives used were replicate 1 of four cell types in original paper [39], and the results of replicate 2 were identical (data not shown). Kernel density estimate plot showing the distribution of log10(half-life + 1). **b** Distribution of half-lives of predicted substrates for each E3. The minimal half-lives in eight experiments of four non-dividing cell types (two replicates per cell type) [39] for substrates were used, and E3s were sorted by the median half-lives of their predicted substrates. **c** Half-life experiments of mutated and wild-type CBX6, the experiment is repeated once (Additional file 5: Table S4). **d** Co-immunoprecipitation experiment between mutated and wild-type CBX6 with SPOP, the experiment is repeated once (Additional file 5: Table S4). Cell lysates were incubated with anti-HA agarose beads, and the immunoprecipitates were analyzed by Western Blot with anti-HA and anti-SPOP antibodies

To evaluate the impact of Degpred degrons on the turnover of proteins, we analyzed half-lives of proteins in non-dividing B cells, natural killer cells, monocytes, and hepatocytes [39]. As shown in Fig. 5a, proteins characterized by dense degrons tend to possess shorter lifespans, which was more significant for proteins with at least five degrons per 1000 AAs. As degrons are more frequent in disordered regions and disorder fraction is positively correlated with degradation rates [19], we analyzed proteins with disorder fractions of 0–10%, 10–30%, 30–100%, respectively, and found that proteins with dense degrons own shorter half-lives in three groups (Additional file 1: Fig. S4). This finding suggested that proteins with more degrons are under stricter regulation of the UPS and are thus degraded faster. To investigate whether different E3s tend to regulate substrates with different half-lives, we compared the half-lives of predicted substrates of different E3s. As shown in Fig. 5b, predicted substrates of TRIM63, βTrCP, NEDD4L, and HUWE1 tend to live shorter, while predicted substrates of TRIM32, FBXL15, PJA1, and FBXL7 tend to possess longer half-lives.

Then, to further verify that predicted degrons prompt protein degradation and mediate E3 binding, we conducted experiments on Chromobox protein homolog 6 (CBX6). CBX6 possessed three Degpred degrons, and segment 269-273 (DARSS) was predicted to be bound by SPOP; CBX6 contains no ELM SPOP motif match. As S is enriched in our generated SPOP motif and is reported to be important in binding with SPOP [13], we mutated DARSS to DARAA. Mutating two AAs can also minimize the impact on protein folding and stability. We transfected wild-type and mutated CBX6 plasmids into HEK293T cells respectively, and cultured cells for 36 h to compare the expression of the transgenes. As shown in Fig. 5c, wild-type CBX6 had much less expression than the mutant, which indicated that mutated CBX6 is more stable in cells. Subsequently, we added cycloheximide to inhibit protein synthesis and found that mutated CBX6 was degraded slower than wild-type CBX6 (Fig. 5c). Next, to test whether DARSS on CBX6 interacts with SPOP, we transfected SPOP and wild-type or mutated CBX6 plasmids into HEK293T cells and conducted co-immunoprecipitation experiments. As shown in Fig. 5d, CBX6 and SPOP co-immunoprecipitated, and mutating CBX6 weakened the interaction with SPOP. These findings indicated that DARSS presenting on CBX6 represents a binding degron of SPOP.

Together, these results demonstrated that E3-degron interactions are principally linked to the control of protein half-lives and different E3s regulate substrates with different degradation rates, which implies that E3 might differ in degradation ability.

### Degron-related mutations on short-lived proteins might drive cancer

Defects in degrons and E3s have been implicated in nearly all hallmarks of cancer [11, 20]. Previous studies found that highly mutated driver regions in cancer contain many known degrons [40], and degron-affecting mutations are positively selected in tumorigenesis [20]. By comparing Degpred degrons with these results, we found that Degpred degrons are enriched in the highly mutated driver regions (Additional file 1: Fig. S5a), including well-known degrons on TP53, MYC, CTNNB1, NFE2L2, and other newly predicted degrons (Additional file 6: Table S5). Besides, motif matches that overlapped with Degpred degrons are under more stringent selection in tumorigenesis than the other motif matches (Additional file 1: Fig. S5b). However, previous studies were limited by using a biased degron set and failed to link degrons to E3s. Here, we investigated alterations of the expanded degron landscape in human cancers and explored the importance of binding E3s in tumorigenesis. We analyzed mutations in 33 cancer types of The Cancer Genome Atlas (TCGA) [41, 42] and cancer driver mutations predicted by CATA-population, CATA-cancer, and Structural clustering [41].

**Fig. 6:**
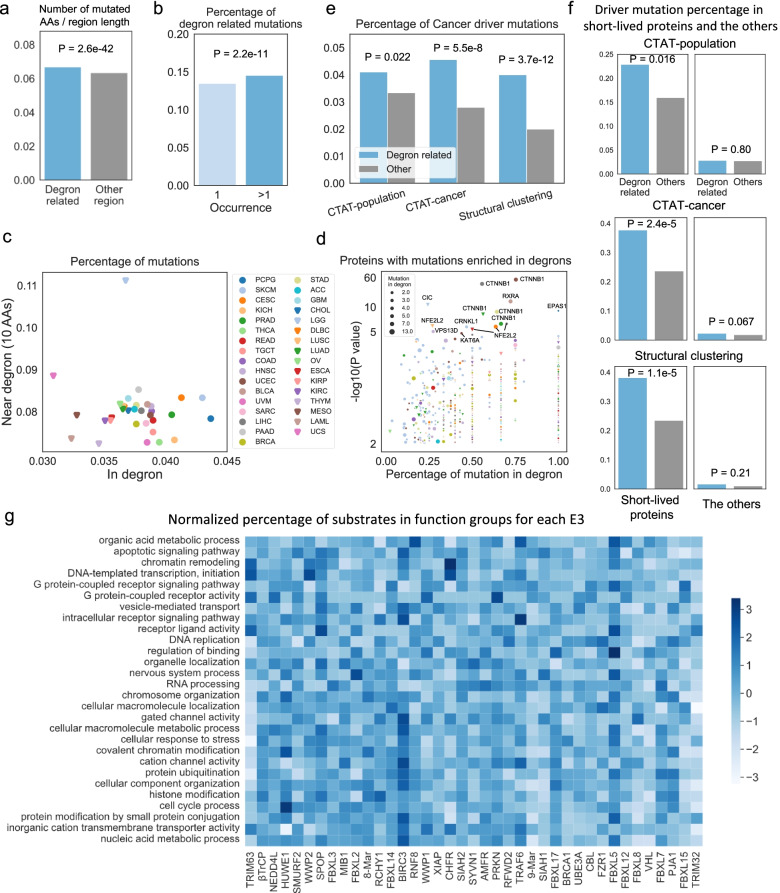
Characterizing TCGA mutations in predicted degrons. **a** Comparison of mutations occurred in degron-related regions (inside and flanking 10 AAs) and other regions, the rates were calculated by dividing the number of AAs with mutations in TCGA in the regions by the number of all AAs of the regions. **b** Comparison of percentage of degron-related mutations between recurrent mutations (> = two tumor samples) and other mutations. **c** Percentage of mutations in degrons and near degrons of 33 cancer types. **d** Proteins with mutations enriched in degrons in 33 cancer types, *P* values were calculated using Fischer’s exact test. Proteins with more than one mutation and *P* value less than 0.01 were shown in the scatter plot. **e** Cancer driver percentage of mutations in degron-related regions and other regions. CATA-population distinguishes pathogenic mutations from benign polymorphisms on a population level; CATA-cancer distinguishes between drivers and passenger somatic mutations; structural clustering leverages information from protein structures to predict drivers. **f** Driver mutation percentage in degron-related regions and other regions of short-lived proteins and the other proteins. **g** Normalized percentage of substrates in each function group for each E3. Percentages of substrates in each function group were normalized to *Z*-scores by rows. The bluer the color, the more substrates in this pathway are regulated by the E3. All *P*-values in this figure were calculated using Fischer’s exact test

By comparing the percentage of AAs with mutations in TCGA in degron-related regions (inside and flanking 10 AAs) and other regions, we found that AAs in degron-related regions are susceptible to mutations in cancer compared with AAs in other regions (Fig. 6a). In addition, we found a higher percentage of recurrent mutations (> = two tumor samples) occur in degron-related regions compared with mutations occurring only once (Fig. 6b). These findings suggested that degron-related mutations are common in human cancer. Then, we investigated degron-related mutations in specific cancer types and proteins. As shown in Fig. 6c, pheochromocytoma and paraganglioma (PCPG), and skin cutaneous melanoma (SKCM) have more mutations in degrons, while brain lower-grade glioma (LGG) contains more mutations near degrons. We next identified hundreds of proteins whose mutations were enriched in degrons in specific cancer types (Fig. 6d, Additional file 6: Table S5). In addition to well-known degron-mutation enriched proteins such as CTNNB1, NFE2L2, and EPAS1 [11, 20], we also identified several proteins rich in degron-mutations that have not been revealed before, such as RXRA in bladder urothelial carcinoma (BLCA), CRNKL1 in skin cutaneous melanoma (SKCM), VPS13D in head and neck squamous cell carcinoma (HNSC), and CIC in LGG. Overall, with the expanded degron landscape, we can explore degron-related mutations in cancer more comprehensively.

Degron-related mutations might interfere with protein degradation and result in abnormal accumulating oncogenes, thus ultimately driving tumorigenesis. We explored whether degron-related mutations tend to act as cancer drivers. Specifically, we focused on recurrent mutations (> = two tumor samples) which are more pathologically significant and tend to occur in degron-related regions (Fig. 6b). Using three different predictors, we found that degron-related mutations are more likely to function as cancer drivers (Fig. 6e). As degron-enriched proteins tend to be short-lived proteins (Fig. 5a) that regulate metabolism, cell proliferation, and differentiation (Additional file 1: Fig. S5c, Additional file 7: Table S6, [43]), we reasoned that degron-related mutations on short-lived proteins might be more pathogenic. To test this hypothesis, we analyzed 1017 short-lived proteins identified by quantitative proteomics in U2OS, HCT116, HEK293T, and RPE1 cell lines [44]. The percentages of driver mutations are significantly higher in short-lived proteins than the other proteins (Fig. 6f), which stressed that short-lived proteins are important in tumorigenesis. Surprisingly, we found that degron-related mutations on short-lived proteins tend to function as cancer drivers compared with other mutations. In contrast, there was no significant difference between these mutations on the other proteins. Further, we used another half-life dataset identified in four non-dividing cell types [39] and compared proteins with the top 1000 shortest half-lives in at least one experiment with the other proteins. We found that degron-related mutations on short-lived proteins in four non-dividing cell types also tend to drive cancer (Additional file 1: Fig. S5d). These results indicated that interfering with the degradation of short-lived proteins is more pathogenic in human cancer, which provides a new perspective for interpreting cancer driver mutations.

Then, we studied E3s in tumorigenesis by analyzing their predicted substrates and binding degrons. We found that approximately two mutations occur in one degron-related region, and the average numbers of mutations in degron-related regions bound by different E3s are comparable (Additional file 1: Fig. S5e). In addition, we found that mutations in degron-related regions bound by SPOP and RFWD2 are more likely to function as cancer drivers (Additional file 1: Fig. S5f), consistent with previous findings that SPOP and RFWD2 regulate the degradation of critical oncogenes [38, 45]. Finally, we analyzed the functions of short-lived substrates of each E3 and identified some well-known functions of these E3s (Fig. 6g), such as CHFR in chromatin remodeling and histone modifications [46], SPOP in histone H3K36 trimethylation and alternative splicing [47], BIRC3 in regulating the caspase and apoptosis pathways [48], and HUWE1 in chromatin modification [49]. Together, these results suggested that E3s regulate different pathways by controlling their substrates, and mutations on degrons bound by different E3s might exert different effects in tumorigenesis.

Finally, we highlighted 19021 degron mutations that alter the charge, hydrophobicity, phosphorylation sites, MoRF regions or predicted protein binding residues [50] of degrons, and 1524 mutations of flanking lysine (Additional file 1: Fig. S5g, Additional file 6: Table S5). These mutations change the properties of degrons and might hinder their function, thus constitute novel potential cancer drivers.

### The web application

**Fig. 7:**
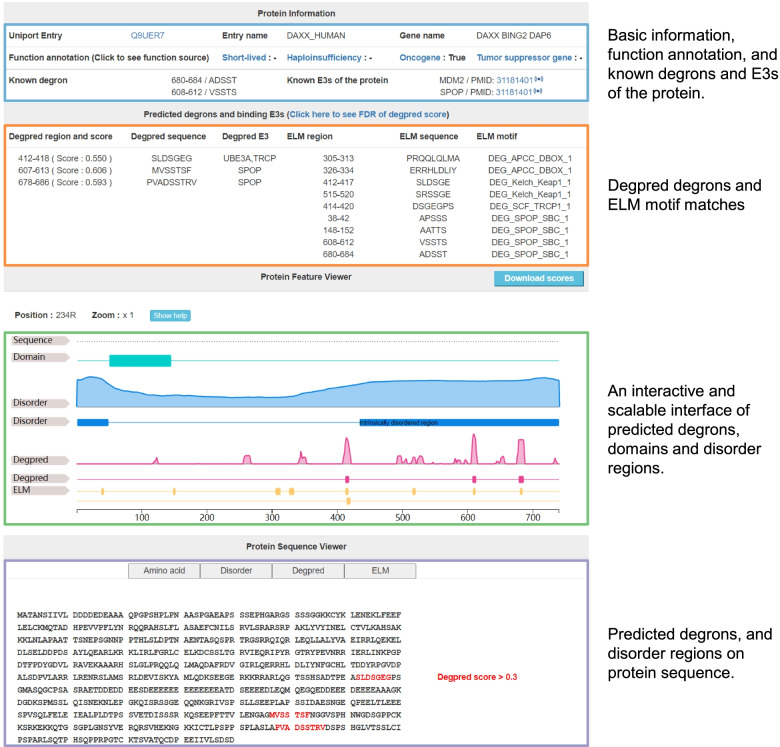
The detail page on our website. The detail page of a protein consists of four parts: (1) basic information, function annotations, and known degrons and E3s; (2) Degpred degrons and ELM motif matches; (3) a feature viewer; and (4) a sequence viewer

A freely available and fully functional website (http://degron.phasep.pro/) has been developed to access the collected and predicted data. Users can search all human proteins on the website according to their gene names and UniProt IDs. The detail page for each protein (Fig. 7) includes four sections: (1) basic information about the protein, haploinsufficiency, short half-life, oncogene, and tumor suppressor gene annotations, known degrons and E3s; (2) Degpred degrons and ELM motif matches of the protein; (3) an interactive and scalable interface [51] showing the regions of domains, intrinsically disorder score and Degpred score along the sequence; and (4) a sequence viewer displaying AAs of regions of interest on the protein sequence. The introduction and summary of the website are described on the “About” page; all data on the website can be freely downloaded on the “Download” page.

## Discussion

Here, we developed a deep learning model to predict hitherto unidentified degrons, allowing for a deeper characterization of the regulatory network involved in protein degradation, both in health and disease. Widely used motif-based methods are limited by few E3 motifs and high false-positive rate. Degpred partially resolves these concerns and captures well-known degron properties. Previous studies showed that machine learning models that integrate multiple protein sequence features perform well in predicting degrons from motif matches [20]; our study suggests that by integrating rich information in the BERT-encoding matrix, deep learning models can achieve comparable performance with feature integrating models, which highlights the power of pre-trained BERT-based models in understanding protein functions. Moreover, our model predicts degrons singly from protein sequence, which needs no time-consuming feature annotation process and can be used to explore the impact of sequence alterations on degron potential.

With the expanded degron landscape and the predicted protein degradation regulatory network, we can study their rules in diseases. In this work, we found that degron-related mutations commonly occur in cancers; we also identified hundreds of proteins rich in degron mutations in different cancer types. Based on the predicted degrons, we can infer possible cancer drivers based on interference of protein degradation instead of recurrence of mutations. Mutated degrons might cause abnormally high expression of proteins and further lead to diseases. We reasoned that mutated degrons on concentration-sensitive proteins like haploinsufficient proteins and short-lived proteins might act as cancer drivers. Our analysis reveals that degron-related mutations on short-lived proteins tend to function as cancer drivers. Thus, our tool provides a new perspective to explain the possible rules of cancer mutations in tumorigenesis and identify possible cancer drivers.

However, our study also has several limitations. (1) In the degron and binding E3 prediction part, first, despite the deep learning method provided, which relies on data available during training, degrons can still not be identified for the overwhelming majority of E3 ligases; second, degrons are under dynamic PTM regulations to control the interaction with E3s, and our method fails to distinguish between modification-dependent and independent degrons; third, our method can only assign degrons to 39 E3s. More experimental verified degrons and ESIs are required to classify degrons with different properties and construct a more comprehensive regulation network. (2) In the experimental part, mutation of SPOP binding degron on CBX6 only weakened the interaction. In a study of SPOP and its substrate DAXX, the authors found that SPOP can bind degrons with one mismatched position of ELM motif [52]. Even though CBX6 possesses no exact ELM SPOP motif match, there are seven potential binding sites on CBX6 with one mismatch with the ELM SPOP motif, which might mediate the interaction with SPOP. Besides, we mutated DARSS to DARAA to minimize the impact on protein folding and stability, mutating two amino acids might not completely block the interaction. (3) In the half-life and cancer mutation parts, we found many interesting results, but we should note that correlation does not mean causality. There exit other factors like disordered regions, MoRFs that may also contribute to these correlations.

Overall, our collected datasets and Degpred model constitute valuable resources to UPS researchers and protein engineers. Our work also suggests novel applications in protein engineering and drug designation. For example, (1) by deleting or adding degrons to proteins, researchers can control protein abundance to regulate specific functions; (2) researchers can mutate the degrons or block E3-degron interactions to upregulate the expression of tumor suppressors [12], which may be a feasible way to treat cancer; and (3) chemists can design PROTAC drugs to link a substrate with a predicted binding E3 to form double bonds between them and achieve higher specificity.

## Conclusions

Our newly designed model provides a powerful and reliable tool for predicting degrons and binding E3s at individual protein and proteome levels. Our work provides novel insights for explaining how a specific subset of driver mutations affect the degradation of proteins and helps bridge the gap between cancer genomics to proteomics. As UPS emerges as a novel therapeutic target in drug discovery and PROTAC drugs show promising effects in clinical trials [53], we anticipate that our work will assist in investigating the pathogenesis of related diseases and provide potential therapeutic targets in the near future.

## Methods

### Data augmentation and degron clustering

Degrons of three and four AAs were symmetrically expanded to five and six AAs respectively to provide more information, and for the convenience of clustering (Additional file 1: Fig. S1a), degrons at the terminus of proteins were expanded in one direction. 128AA-peptides used to train the model were randomly sampled around degrons from original protein sequences. For degrons near or at the terminus of proteins, we added “X” to the terminus of the sequence to ensure that degrons were evenly distributed on 128AA-peptides. GibbsCluster [28] was used for degrons clustering with parameters: number of clusters = 5, motif length = 5, default values were used for other parameters. Five clusters were used because five-fold cross-validation is widely used in evaluating machine learning models.

### Model architecture, training, and test processes

Models were built using Pytorch 1.4 (https://pytorch.org/). The input protein sequence of L AAs was embedded using the TAPE BERT-based model [27] (https://github.com/songlab-cal/tape) to a 768 * (L+2) feature matrix or embedded to a 20 * L one-hot encoding matrix. The embedded matrix was passed to bidirectional long short-term memory (biLSTM) layers and fully connected layers. The sigmoid function was used in the final node to ensure the output for each AA was always between 0 and 1. The BERT-based model contains 230,929 trainable parameters, while the one-hot model contains 243,729 trainable parameters.

AAs in degrons were labeled as 1, AAs in the other regions were labeled as 0, and flanking three AAs of degrons were not used in the training and test processes, as these AAs may contribute to E3 binding as well, but were not verified in experiment. In the training process, a dropout rate of 0.3 was employed to avoid overfitting, the batch size was 32. The weighted cross-entropy function was utilized as the loss function, defined as follows:

$$Loss=-\frac{1}{n^2}\sum \left(\left(n-{n}_{deg}\right)y\log \left({y}_{pred}\right)+{n}_{deg}\left(1-y\right)\log \left(1-{y}_{pred}\right)\right)$$

where *n* is the number of all AAs, *n*_*deg*_ is the number of AAs in degrons, *y* is the label, and *y*_*pred*_ is the predicted value. The training process stopped when the loss did not decrease in the following five epochs. The Adam optimizer with a learning rate of 0.0003 was used to update parameters.

In the test process, 0.5 was used as the cut-off to classify AAs to be in degrons or not. Models were evaluated using the area under the receiver operating characteristic curve (AUC), precision-recall curves, and the following scores:

$$\mathrm{precision}=\frac{TP}{TP+ FP}$$

$$\mathrm{recall}=\frac{TP}{TP+ FN}$$

$$\mathrm{FDR}=\frac{FP}{TP+ FP}$$

$$\mathrm{accuracy}=\frac{TP+ TN}{TP+ FP+ TN+ FN}$$

where TP represents the number of AAs in degrons identified correctly, TN represents the number of AAs in other regions identified correctly, FN denotes the number of AAs in degrons identified incorrectly, FP represents the number of AAs in other regions identified incorrectly.

### Comparing the BERT-based model with Motif_RF and MoRFchibi

For the TAPE BERT-based model, the inputs were 128AA-peptides with motif matches located at the center; one 128AA-peptide was generated for each motif match (no data augmentation in this comparison). The predicted scores for AAs in the motif match were averaged to represent the BERT-based model score of the motif match, the average scores were used to calculate cross-entropy loss in the training stage and evaluated in the test stage. Motif_RF was built by the scikit-learn package of python with same parameters as the original paper [20]. The input features for Motif_RF were directly downloaded from the supplementary data of the original paper [20]. The average MoRFchibi score of AAs in the motif match was used as the MoRFchibi score of the motif match.

Our collected known degrons possessing feature annotations in the supplementary data of the original Motif_RF paper [20] constituted the positive dataset (only retaining one degron for the same degrons on different isoforms of one gene); the negative dataset contains motif matches randomly selected from proteins without known degrons. The ratio of positive to negative samples is 1:5. Fivefold cross-validation was used to calculate the AUC of TAPE BERT-based model and Motif_RF; AUC of MoRFchibi was directly calculated on all motif matches in positive and negative datasets.

### Degron prediction

To predict degrons from protein sequence, the average of outputs from models 1–5 was calculated and 0.3 was set as the cut-off. Two predicted degrons with a distance of fewer than 4 AAs were merged, and degrons shorter than 3 AAs were discarded. Due to the O(L2) space complexity of BERT-based models, handling the full length of long proteins requires high computational power. Thus, for proteins shorter than 1000 AAs, the full proteins were used; for proteins longer than 1000 AAs, the proteins were evenly split into ceil(L/1000) parts and predicted separately, where *L* is the length of proteins, and ceil means to round up the value upward to the smallest integer not less than it.

### Properties comparison

MoRFchibi, ASAquick and predicted protein binding residue (SCRIBER) scores were downloaded from DescribePROT [50]. MoRF regions and predicted protein binding residues in Additional file 1: Fig. S5g were residues with top 5% highest score in the human proteome. ESpritz-Disport [34] with default parameters was used to predict disordered regions. Phosphorylation sites were downloaded from PhosphoSitePlus [31] (2021.01.19); the other PTM sites were downloaded from dbPTM [32] (2020.12.31).

Degpred degrons in Fig. 3b–g were predicted at the cut-off of 0.3 with an FDR of 0.512 at positive: negative = 1:20 (Additional file 1: Fig. S2a). Predicted degrons of Motif_RF in Fig. 3b–g were motif matches in the supplementary data of the original paper [20] with Prob_DEGRON larger than 0.842, which attained the same FDR at positive: negative = 1:20 with our method. The random peptides used in Fig. 3b–d and Additional file 1: Fig. S2c were peptides of 10 AAs sampled from the human proteome. The number of peptides sampled from a protein of length L is ceil(L/1000), where ceil means to round the value upward to the smallest integer not less than it. About 33,000 random peptides were sampled from the human proteome.

### ESI dataset collection

Nine hundred sixty-five related papers were obtained by searching PubMed with keywords: (E3[Title] OR E3s[Title]) AND (substrate[Title/Abstract] OR substrates[Title/Abstract]). Twenty-seven of 965 papers were retained after the manual screening. Two thousand two hundred seventy-two nonredundant ESIs between 56 human E3s and 1863 substrates were extracted from these studies, and most of them were identified from high throughput experiments. In addition, Chen et al. [22] had collected 1790 nonredundant known ESIs from BioGrid [54], E3Net [55], hUbiquitome [56], and UniProt [23]; these ESIs were included as well. In total, we obtained 3766 nonredundant ESIs between 307 human E3s and 2713 substrates (Additional file 4: Table S3).

### Motif calculation and matching

As known substrates from public databases are more reliable than manual high-throughput substrates, for E3s with at least ten known substrates, only known substrates were used to calculate motifs; for E3s with less than ten known substrates, both known substrates and high-throughput substrates were used. Degrons and flanking three AAs on both sides were used in motif calculation and matching. GibbsCluster [28] was employed to align these expanded degrons and remove outliers. Given the length of known degrons, the motif length was set to 5-8 AAs, and four motifs with lengths of 5-8 would be generated for each E3. A trash cluster was used to remove outliers, and the trash cluster threshold was set to 4. Alignment cores with lengths of 5-8 generated by GibbsCluster [28] were used to construct four position-specific score matrixes (PSSMs) for each E3. For a PSSM, *S*_*i*, *a*_ represents the score of amino acid *a* on position *i*, and was calculated as:

$${S}_{i,a}={\log}_2\left(\frac{\left({C}_{i,a}+1\right)/\left(N+20\right)}{F_a}\right)$$

where *C*_*i*, *a*_ is the number of amino acid *a* on position *i* in alignment cores, *N* is the number of alignment cores, and *F*_*a*_ is the frequency of amino acid *a* in the human proteome.

For an equal-length peptide with a PSSM, the matching score was calculated by adding scores of all AAs in the peptide; for a peptide longer than a PSSM, the peptide was slid across with the PSSM, and the highest matching score among all positions was used as the matching score; for a peptide shorter than a PSSM, no matching score was provided. To calculate the cut-off for a PSSM, we reversed it and used the reversed PSSM to score 1,000,000 equal-length peptides randomly sampled from human proteome, the top 1/2000 score was used as the cut-off for the PSSM. For each E3, the ability of four PSSMs to capture known substrates was evaluated, and the PSSM with higher recall rate was selected as the motif representing the E3.

Seven of 39 E3s with calculated motifs possess ELM motifs (SPOP: DEG_SPOP_SBC_1, SIAH1 and SIAH2: DEG_SIAH_1, βTrCP: DEG_SCF_TRCP1_1, VHL: DEG_ODPH_VHL_1, RFWD2: DEG_COP1, FZR1: DEG_APCC_KENBOX_2). Four of 16 E3s without calculated motifs possess ELM motifs (SKP2: DEG_SCF_SKP2-CKS1_1, MDM2: DEG_MDM2_SWIB_1, FBXW7: DEG_SCF_FBW7_1 and DEG_SCF_FBW7_2, CDC20: DEG_APCC_DBOX_1).

### Public data and gene ontology enrichment

For SPOP overexpressed ubiquitylome and proteome data [38], only data of replicate 1 was compared, and the results of replicate 2 were identical (data not shown). The half-lives used in Fig. 5a and Fig. S4 were replicate 1 of four cell types [39], and the results of replicate 2 were identical (data not shown). TCGA mutation data were downloaded from https://gdc.cancer.gov/about-data/publications/mc3-2017 and processed according to ref. [41]; cancer driver mutation data were downloaded from https://gdc.cancer.gov/about-data/publications/pancan-driver.

Gene ontology enrichment was conducted using Cytoscape package clueGO (version 2.5.8, https://apps.cytoscape.org/apps/cluego). The detailed parameters are available in Additional file 7: Table S6.

### Cell culture and transfection

HEK293T cells were grown in DMEM (Biological Industries) supplemented with 10% fetal bovine serum. Cells were cultured in a 37 °C/5% CO2 incubator. Plasmids were transfected into cells with PEI (Hannothch), the culture medium was changed after 6 h of transfection, and cells were maintained for another 36 h.

### Plasmids and antibodies

Wide type and mutated CBX6 plasmids were synthesized by GENEWIZ. HA-tagged wild-type and mutated CBX6 were cloned into plvx-IRES-zsgreen. The plvx-empty and plvx-myc-SPOP were provided by Dr. Hong Wu lab.

Anti-HA: Santa Cruz Biotechnology sc-7392; Anti-GAPDH: Cell Signaling Technology D16H11; Anti-SPOP was homemade in Dr. Hong Wu lab.

### Cycloheximide chasing assay

Wide type or mutated CBX6 plasmids were transfected into HEK293T cells. Cycloheximide (MCE 100 μg/ml) was added into the medium after incubation and cells were collected at different time points. Cells were washed with PBS, lysed in RIPA buffer (50 mM Tris-Cl pH 7.4, 150 mM NaCl, 1% TrintonX-100, 1% sodium deoxycholate, 1% SDS, plus protease inhibitor cocktails (Thermo Scientific)). Cell lysates were analyzed by Western Blot with indicated antibodies.

### Co-immunoprecipitation assay

Myc-SPOP and wide type or mutated HA-CBX6 were transfected into HEK293T cells. Cell lysates were prepared 48 h after transfected in Sucrose-NP40 lysis buffer (25 mM Tris-Cl pH 7.5, 150 mM NaCl, 5 mM MgCl2, 1 mM DTT, 1 mM PMSF, 10 mM NaF, 1 mM NaVO3, 2 mM EDTA, 0.25 M Sucrose and 0.5% NP40 and protease inhibitors (Roche 04906837001)) for 30 min on ice. Cell lysates were spun at 15,000 rpm for 15 min at 4 °C, and the supernatants were incubated with pre-washed anti-HA (Thermo 26182) agarose beads for 4 h in the cold room. The immunoprecipitates were collected by centrifugation and washed four times with wash buffer (150 mM NaCl, 25 mM Tris-Cl pH 7.5, 0.1% NP40, 5 mM MgCl_2_, 1 mM DTT, 1 mM PMSF). Precipitations were analyzed by Western blot after 90 °C 10 min boiled in 1 × loading buffer (0.2 M Tris-Cl pH 6.8, 0.02 g/mL SDS, 1 mg/mL Bromophenol Blue, 10% glycerinum, 1% β-Me).

### Western Blot

Cell lysates were boiled in protein loading buffer and centrifuged at 14,000g. The protein supernatants were subjected to 10% SDS-PAGE. Proteins were then transferred into 0.45 μm PVDF membranes (Millpore), and the membrane were blocked in 5% BSA for 1 h and were incubated with primary antibodies overnight at 4 °C. After washing with TBST (0.5% Tween-20), membranes with protein were incubated with secondary antibody for 2 h at room temperature. After washing with TBST (0.5% Tween-20), proteins of interest were visualized using the enhanced chemiluminescence system (Thermo). Uncropped western blots were provided in Additional file 8.

## Supplementary Information

**Additional file 1: Figure S1**. Architecture of the model and comparison with other predictors. **Figure S2**. Cut-off determination, comparison with GPS experiment, and PTMs in degrons. **Figure S3**. Statistics of collected and predicted ESI datasets, and motifs for HECT E3s. **Figure S4**. Half-lives of proteins with different disorder fractions and degron density. **Figure S5**. Functional analysis of degron-related mutations and E3s.

**Additional file 2: Table S1**. Known degrons from ELM and three previous works, and Degpred degrons on UniProt human reviewed proteins with 0.3 as the cutoff.

**Additional file 3: Table S2**. Known ESIs and manual collected ESIs.

**Additional file 4: Table S3**. Calculated motifs and predicted ESIs.

**Additional file 5: Table S4**. Individual values for figures where the number of independent replicates is less than 6.

**Additional file 6: Table S5**. Degrons in driver regions, proteins rich in degron mutations, and novel potential driver mutations in Degpred degrons.

**Additional file 7: Table S6**. Enriched function groups of short-lived proteins.

**Additional file 8.** Uncropped western blots.

## Data Availability

Both collected and predicted data are provided as additional files and can be downloaded from http://degron.phasep.pro/. The code is available at 10.5281/zenodo.6722109 or https://github.com/CHAOHOU-97/degpred. All data generated or analyzed during this study are included in this published article, its supplementary information files and publicly available repositories.

## Abbreviations

AA: Amino acid
AP-MS: Affinity purification mass spectrometry
AUC: The area under the receiver operating characteristic curve
BLCA: Bladder Urothelial Carcinoma
CBX6: Chromobox protein homolog 6
ChenESI: A E3-substrate predictor developed by Chen et al.
E3: E3 ubiquitin ligase
ESI: E3-substrate interaction
GPS: Global Protein Stability
HNSC: Head and Neck Squamous Cell Carcinoma
LGG: Brain Lower Grade Glioma
MoRF: Molecular recognition feature
Motif_RF: A degron predictor developed by Martínez-Jiménez et al.
PCPG: Pheochromocytoma and paraganglioma
PSSM: Position-specific score matrix
PTM: Post-translational modification
SKCM: Skin cutaneous melanoma
TCGA: The Cancer Genome Atlas
UPS: Ubiquitin-proteasome system
Ub: Ubiquitin

## References

[1] Meszaros B, Kumar M, Gibson TJ, Uyar B, Dosztanyi Z (2017). Degrons in cancer. Sci Signal.

[2] Ciechanover A (2017). Intracellular protein degradation: From a vague idea thru the lysosome and the ubiquitin-proteasome system and onto human diseases and drug targeting. Best Pract Res Clin Haematol.

[3] Collins GA, Goldberg AL (2017). The logic of the 26S proteasome. Cell.

[4] Buchberger A, Bukau B, Sommer T (2010). Protein quality control in the cytosol and the endoplasmic reticulum: brothers in arms. Mol Cell.

[5] Kwon YT, Ciechanover A (2017). The ubiquitin code in the ubiquitin-proteasome system and autophagy. Trends Biochem Sci.

[6] Li W, Bengtson MH, Ulbrich A, Matsuda A, Reddy VA, Orth A, Chanda SK, Batalov S, Joazeiro CA (2008). Genome-wide and functional annotation of human E3 ubiquitin ligases identifies MULAN, a mitochondrial E3 that regulates the organelle's dynamics and signaling. PLoS One.

[7] Guharoy M, Bhowmick P, Sallam M, Tompa P (2016). Tripartite degrons confer diversity and specificity on regulated protein degradation in the ubiquitin-proteasome system. Nat Commun.

[8] Malhis N, Jacobson M, Gsponer J (2016). MoRFchibi SYSTEM: software tools for the identification of MoRFs in protein sequences. Nucleic Acids Res.

[9] Van Roey K, Dinkel H, Weatheritt RJ, Gibson TJ, Davey NE (2013). The switches.ELM resource: a compendium of conditional regulatory interaction interfaces. Sci Signal.

[10] Koren I, Timms RT, Kula T, Xu Q, Li MZ, Elledge SJ (2018). The eukaryotic proteome is shaped by E3 ubiquitin ligases targeting C-terminal degrons. Cell.

[11] Tokheim C, Wang X, Timms RT, Zhang B, Mena EL, Wang B, Chen C, Ge J, Chu J, Zhang W (2021). Systematic characterization of mutations altering protein degradation in human cancers. Mol Cell.

[12] Ruan H, Yu C, Niu X, Zhang W, Liu H, Chen L, Xiong R, Sun Q, Jin C, Liu Y (2020). Computational strategy for intrinsically disordered protein ligand design leads to the discovery of p53 transactivation domain I binding compounds that activate the p53 pathway. Chem Sci.

[13] Zhang Q, Shi Q, Chen Y, Yue T, Li S, Wang B, Jiang J (2009). Multiple Ser/Thr-rich degrons mediate the degradation of Ci/Gli by the Cul3-HIB/SPOP E3 ubiquitin ligase. Proc Natl Acad Sci U S A.

[14] Ella H, Reiss Y, Ravid T. The hunt for degrons of the 26S proteasome. Biomolecules. 2019;9(6):230. 10.3390/biom9060230 PMC6628059 31200568

[15] Iconomou M, Saunders DN (2016). Systematic approaches to identify E3 ligase substrates. Biochem J.

[16] Kumar M, Gouw M, Michael S, Sámano-Sánchez H, Pancsa R, Glavina J, Diakogianni A, Valverde JA, Bukirova D, Čalyševa J (2019). ELM—the eukaryotic linear motif resource in 2020. Nucleic Acids Res.

[17] Timms RT, Zhang Z, Rhee DY, Harper JW, Koren I, Elledge SJ. A glycine-specific N-degron pathway mediates the quality control of protein N-myristoylation. Science. 2019;365(6448):eaaw4912. 10.1126/science.aaw4912 PMC7090375 31273098

[18] Fishbain S, Inobe T, Israeli E, Chavali S, Yu H, Kago G, Babu MM, Matouschek A (2015). Sequence composition of disordered regions fine-tunes protein half-life. Nat Struct Mol Biol.

[19] van der Lee R, Lang B, Kruse K, Gsponer J, Sanchez de Groot N, Huynen MA, Matouschek A, Fuxreiter M, Babu MM (2014). Intrinsically disordered segments affect protein half-life in the cell and during evolution. Cell Rep.

[20] Martínez-Jiménez F, Muiños F, López-Arribillaga E, Lopez-Bigas N, Gonzalez-Perez A (2019). Systematic analysis of alterations in the ubiquitin proteolysis system reveals its contribution to driver mutations in cancer. Nat Can.

[21] Wang X, Li Y, He M, Kong X, Jiang P, Liu X, et al. UbiBrowser 2.0: a comprehensive resource for proteome-wide known and predicted ubiquitin ligase/deubiquitinase-substrate interactions in eukaryotic species. Nucleic Acids Res. 2021;50(D1):D719–D728. 10.1093/nar/gkab962 PMC8728189 34669962

[22] Chen D, Liu X, Xia T, Tekcham DS, Wang W, Chen H, Li T, Lu C, Ning Z, Liu X (2019). A multidimensional characterization of E3 ubiquitin ligase and substrate interaction network. iScience.

[23] UniProt C (2019). UniProt: a worldwide hub of protein knowledge. Nucleic Acids Res.

[24] Liu J, Tokheim C, Lee JD, Gan W, North BJ, Liu XS, Pandolfi PP, Wei W (2021). Genetic fusions favor tumorigenesis through degron loss in oncogenes. Nat Commun.

[25] Vig J, Madani A, Varshney LR, Xiong C, Socher R, Rajani NF. Bertology meets biology: interpreting attention in protein language models. arXiv preprint arXiv:2006.15222. 2020.

[26] Rives A, Meier J, Sercu T, Goyal S, Lin Z, Liu J, et al. Biological structure and function emerge from scaling unsupervised learning to 250 million protein sequences. Proc Natl Acad Sci U S A. 2021;118(15):e2016239118. 10.1073/pnas.2016239118 PMC8053943 33876751

[27] Rao R, Bhattacharya N, Thomas N, Duan Y, Chen X, Canny J, Abbeel P, Song YS (2019). Evaluating protein transfer learning with TAPE. Adv Neural Inf Proces Syst.

[28] Andreatta M, Alvarez B, Nielsen M (2017). GibbsCluster: unsupervised clustering and alignment of peptide sequences. Nucleic Acids Res.

[29] Low TY, Peng M, Magliozzi R, Mohammed S, Guardavaccaro D, Heck AJ (2014). A systems-wide screen identifies substrates of the SCFbetaTrCP ubiquitin ligase. Sci Signal.

[30] Coyaud E, Mis M, Laurent EM, Dunham WH, Couzens AL, Robitaille M, Gingras AC, Angers S, Raught B (2015). BioID-based identification of Skp Cullin F-box (SCF)beta-TrCP1/2 E3 ligase substrates. Mol Cell Proteomics.

[31] Hornbeck PV, Kornhauser JM, Latham V, Murray B, Nandhikonda V, Nord A, Skrzypek E, Wheeler T, Zhang B, Gnad F (2019). 15 years of PhosphoSitePlus(R): integrating post-translationally modified sites, disease variants and isoforms. Nucleic Acids Res.

[32] Huang KY, Lee TY, Kao HJ, Ma CT, Lee CC, Lin TH, Chang WC, Huang HD (2019). dbPTM in 2019: exploring disease association and cross-talk of post-translational modifications. Nucleic Acids Res.

[33] Rechsteiner M, Rogers SW (1996). PEST sequences and regulation by proteolysis. Trends Biochem Sci.

[34] Walsh I, Martin AJ, Di Domenico T, Tosatto SC (2012). ESpritz: accurate and fast prediction of protein disorder. Bioinformatics.

[35] Faraggi E, Zhou Y, Kloczkowski A (2014). Accurate single-sequence prediction of solvent accessible surface area using local and global features. Proteins.

[36] Ingham RJ, Gish G, Pawson T (2004). The Nedd4 family of E3 ubiquitin ligases: functional diversity within a common modular architecture. Oncogene.

[37] Ingham RJ, Colwill K, Howard C, Dettwiler S, Lim CS, Yu J, Hersi K, Raaijmakers J, Gish G, Mbamalu G (2005). WW domains provide a platform for the assembly of multiprotein networks. Mol Cell Biol.

[38] Theurillat JP, Udeshi ND, Errington WJ, Svinkina T, Baca SC, Pop M, Wild PJ, Blattner M, Groner AC, Rubin MA (2014). Prostate cancer. ubiquitylome analysis identifies dysregulation of effector substrates in SPOP-mutant prostate cancer. Science.

[39] Mathieson T, Franken H, Kosinski J, Kurzawa N, Zinn N, Sweetman G, Poeckel D, Ratnu VS, Schramm M, Becher I (2018). Systematic analysis of protein turnover in primary cells. Nat Commun.

[40] Meszaros B, Hajdu-Soltesz B, Zeke A, Dosztanyi Z. Mutations of intrinsically disordered protein regions can drive cancer but lack therapeutic strategies. Biomolecules. 2021;11(3):381. 10.3390/biom11030381 PMC8000335 33806614

[41] Bailey MH, Tokheim C, Porta-Pardo E, Sengupta S, Bertrand D, Weerasinghe A, Colaprico A, Wendl MC, Kim J, Reardon B (2018). Comprehensive characterization of cancer driver genes and mutations. Cell.

[42] Ellrott K, Bailey MH, Saksena G, Covington KR, Kandoth C, Stewart C, Hess J, Ma S, Chiotti KE, McLellan M (2018). Scalable open science approach for mutation calling of tumor exomes using multiple genomic pipelines. Cell Syst.

[43] Bindea G, Mlecnik B, Hackl H, Charoentong P, Tosolini M, Kirilovsky A, Fridman W-H, Pagès F, Trajanoski Z, Galon J (2009). ClueGO: a Cytoscape plug-in to decipher functionally grouped gene ontology and pathway annotation networks. Bioinformatics (Oxford, England).

[44] Li J, Cai Z, Vaites LP, Shen N, Mitchell DC, Huttlin EL, et al. Proteome-wide mapping of short-lived proteins in human cells. Mol Cell. 2021;81(22):4722–35.e5. 10.1016/j.molcel.2021.09.015 PMC8892350 34626566

[45] Zhang Y, Yokoyama S, Herriges JC, Zhang Z, Young RE, Verheyden JM, Sun X (2016). E3 ubiquitin ligase RFWD2 controls lung branching through protein-level regulation of ETV transcription factors. Proc Natl Acad Sci U S A.

[46] Gagné JP, Pic E, Isabelle M, Krietsch J, Ethier C, Paquet E, Kelly I, Boutin M, Moon KM, Foster LJ (2012). Quantitative proteomics profiling of the poly(ADP-ribose)-related response to genotoxic stress. Nucleic Acids Res.

[47] Zhu K, Lei PJ, Ju LG, Wang X, Huang K, Yang B, Shao C, Zhu Y, Wei G, Fu XD (2017). SPOP-containing complex regulates SETD2 stability and H3K36me3-coupled alternative splicing. Nucleic Acids Res.

[48] Fakiruddin KS, Lim MN, Nordin N, Rosli R, Zakaria Z, Abdullah S. Targeting of CD133+ cancer stem cells by mesenchymal stem cell expressing TRAIL reveals a prospective role of apoptotic gene regulation in non-small cell lung cancer. Cancers. 2019;11(9):1261. 10.3390/cancers11091261 PMC6770521 31466290

[49] Huang YL, Zhang PF, Hou Z, Fu Q, Li MX, Huang DL, et al. Ubiquitome analysis reveals the involvement of lysine ubiquitination in the spermatogenesis process of adult buffalo (Bubalus bubalis) testis. Biosci Rep. 2020;40(6):BSR20193537. 10.1042/BSR20193537 PMC7298129 32469046

[50] Zhao B, Katuwawala A, Oldfield CJ, Dunker AK, Faraggi E, Gsponer J, Kloczkowski A, Malhis N, Mirdita M, Obradovic Z (2020). DescribePROT: database of amino acid-level protein structure and function predictions. Nucleic Acids Res.

[51] Paladin L, Schaeffer M, Gaudet P, Zahn-Zabal M, Michel PA, Piovesan D, Tosatto SCE, Bairoch A (2020). The Feature-Viewer: a visualization tool for positional annotations on a sequence. Bioinformatics.

[52] Bouchard JJ, Otero JH, Scott DC, Szulc E, Martin EW, Sabri N, Granata D, Marzahn MR, Lindorff-Larsen K, Salvatella X (2018). Cancer mutations of the tumor suppressor SPOP disrupt the formation of active, phase-separated compartments. Mol Cell.

[53] Paiva SL, Crews CM (2019). Targeted protein degradation: elements of PROTAC design. Curr Opin Chem Biol.

[54] Oughtred R, Stark C, Breitkreutz BJ, Rust J, Boucher L, Chang C, Kolas N, O'Donnell L, Leung G, McAdam R (2019). The BioGRID interaction database: 2019 update. Nucleic Acids Res.

[55] Han Y, Lee H, Park JC, Yi GS (2012). E3Net: a system for exploring E3-mediated regulatory networks of cellular functions. Mol Cell Proteomics.

[56] Du Y, Xu N, Lu M, Li T. hUbiquitome: a database of experimentally verified ubiquitination cascades in humans. Database (Oxford). 2011;2011:bar055. 10.1093/database/bar055 PMC3228279 22134927

