# Supplementary material for: Systematic prediction of degrons and E3 ubiquitin ligase binding via deep learning
Source: BMC Biol. 2022 Jul 14;20:162. doi: 10.1186/s12915-022-01364-6 (PMC9281121; doi:10.1186/s12915-022-01364-6)
Supplement: Supplementary file 1 — Additional file 1: Figure S1. Architecture of the model and comparison with other predictors. Figure S2. Cut-off determination, comparison with GPS experiment, and PTMs in degrons. Figure S3. Statistics of collected and predicted ESI datasets, and motifs for HECT E3s. Figure S4. Half-lives of proteins with different disorder fractions and degron density. Figure S5. Functional analysis of degron-related mutations and E3s. [file 12915_2022_1364_MOESM1_ESM.pdf]

Additional file 1: Fig. S1-S5

Systematic Prediction of Degrons and E3 Ubiquitin Ligase Binding via Deep Learning

Chao Hou[1,2], Yuxuan Li[1,2], Mengyao Wang[3,4], Hong Wu[3,4,5], Tingting Li[1,2,*]

[1]Department of Biomedical Informatics, School of Basic Medical Sciences, Peking University Health Science Center, Beijing 100191, China.

[2]Key Laboratory for Neuroscience, Ministry of Education/National Health Commission of China, Peking University, Beijing 100191, China.

[3]The MOE Key Laboratory of Cell Proliferation and Differentiation, School of Life Sciences, Peking University, Beijing 100871, China.

[4]Peking-Tsinghua Center for Life Sciences, Beijing, China.

[5]Peking University Institute of Hematology, National Clinical Research Center for Hematologic Disease, Beijing, China.

*Corresponding Author: litt@hsc.pku.edu.cn.

Supplementary Figure legends

Fig. S1 Architecture of the model and comparison with other predictors. a Distribution of length of degrons and expanded degrons. **b** Detailed architecture of the TAPE BERT-based degron prediction model. **c** Performances of the BERT-based model, Motif_RF and MoRFchibi in predicting degrons from motif matches, curves of the BERT-based model, Motif_RF were averages in five-fold cross-validation, MoRFchibi was directly evaluated on all positive and negative motif matches. **d** Averaged receiver operating characteristic curves of the BERT-based and one-hot models. **e** Evaluation scores of the BERT-based model and one-hot model in predicting five clusters of degrons, AUC and recall were measured at positive to negative ratio equal to that in the training set, negative samples were AAs randomly selected from proteins possessing test degrons. **f** Averaged precision-recall curves of BERT-based models at different positive to negative ratios. **g** Detailed architecture of the one-hot degron prediction model. **f** Averaged precision-recall curves of one-hot models at different positive to negative ratios.

Fig. S2 Cut-off determination, comparison with GPS experiment and PTMs in degrons. a FDR and recall of Degpred at different cut-offs with positive: negative=1:20, negative samples were AAs randomly selected from proteins possessing degrons. **b** Distribution of terminus located known and Degpred degrons in GPS N-end and C-end experiments. Known and Degpred degrons located within 23 AAs distant from N-end and C-end were compared, consistent with the peptide length used in GPS experiments. The smaller the number of the bin, the stronger the ability of peptides of the bin to induce reporter protein degradation. **c** Enrichment of PTM sites in Degpred degrons, the bars show the fold change of ratios of modified AAs in Degpred degrons and random peptides from the human proteome. P-values were calculated using Fischer's exact test.

Fig. S3 Statistics of collected and predicted ESI datasets and motifs for HECT E3s. a Number of substrates for each E3 (left) and number of E3s for each substrate (right) in our collected ESI dataset. **b** Seqlogos of generated motifs of four HECT E3s: WWP1, WWP2, SMURF2, NEDD4L. **c** Precision and recall rates for 55 E3s. Precisions were calculated at positive: negative=1:20. Recall rates measured the percentage of known substrates that can be predicted using calculated motifs. The raw numbers of this figure were provided in Supplementary Table 3. **d** Comparison of ChenESI and generated motifs in predicting substrates of SPOP and FZR1 in manually collected ESIs of Ubibrowser2.0. ChenESI predicted 1186 SPOP substrates and 2020 FZR1 substrates, we selected top 890 SPOP substrates and top 1179 FZR1 substrates of Degpred to get the same FDR with ChenESI. **e** Number of substrates for each E3 (upper) and number of E3s for each substrate (lower) in the predicted ESI dataset.

Fig. S4 Half-lives of proteins with different disorder fractions and degron density. Distribution of protein half-lives in four non-dividing cell types, proteins were divided into three groups with disorder fractions 0-10%, 10-30%, 30-100%, numbers of proteins were shown on the right. The

half-lives used were replicate 1 of four cell types in original paper, and the results of replicate 2 were identical (data not shown). Kernel density estimate plots showing the distribution of log10(half-life + 1).

Fig. S5 Functional analysis of degron-related mutations and E3s. a Percentage of Degpred degron AAs in driver regions and other regions. P-value was calculated using Fischer's exact test. **b** Distribution of -log(P-value) calculated by SMDeg and FMDeg of motif matches overlapped with Degpred degrons and not overlapped. SMDeg probes the over-representation of missense mutations in degrons with respect to the number inferred from the distribution of all missense mutations observed in the protein. FMDeg computes the deviation of the average functional impact of missense mutations in degrons from the expected impact. P-values were calculated using two side T-test. **c** Enriched function groups of short-lived proteins. **d** Percentage of driver mutations in degron-related regions and other regions of short-lived proteins and the other proteins in four non-dividing cell types. P-values were calculated using Fischer's exact test. **e** Average number of TCGA mutations occurring in degron-related regions bound by different E3s. **f** Percentage of driver mutations in degron-related regions bound by different E3s. **g** Percentage of mutations that alter the charge, hydrophobicity, phosphorylation sites, MoRF regions, predicted protein binding residues or lysine residues.

a

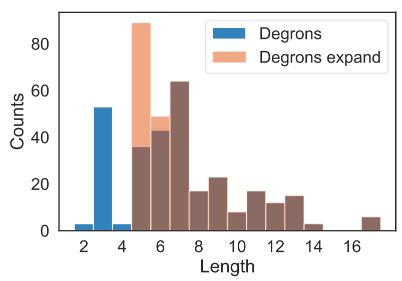

c

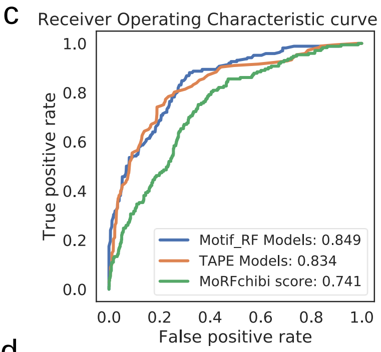

f

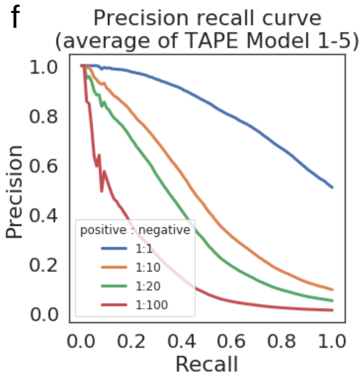

b

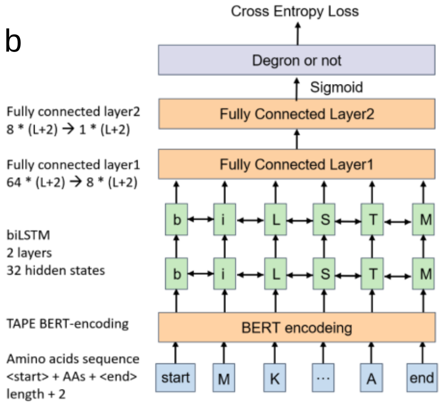

d

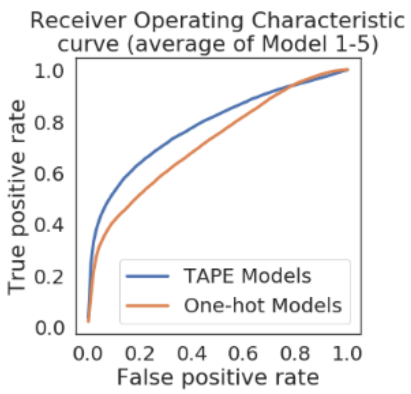

g

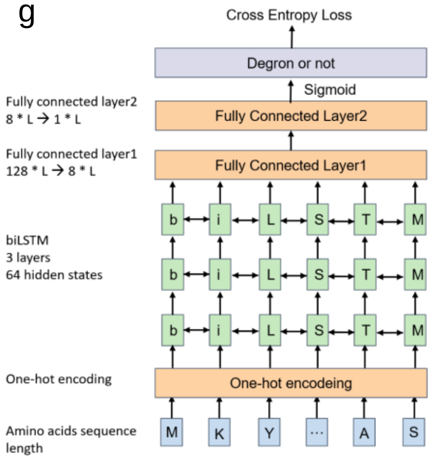

e

	AUC	Recall	Accuracy	Precision	FDR
TAPE Model 1	0.8126	0.433	0.704 / 0.947	0.944 / 0.443	0.056 / 0.557
One-hot Model 1	0.7855	0.511	0.71 / 0.897	0.85 / 0.234	0.15 / 0.766
TAPE Model 2	0.7836	0.403	0.682 / 0.937	0.913 / 0.354	0.087 / 0.646
One-hot Model 2	0.7613	0.359	0.653 / 0.917	0.871 / 0.248	0.129 / 0.752
TAPE Model 3	0.6911	0.235	0.605 / 0.943	0.904 / 0.357	0.096 / 0.643
One-hot Model 3	0.6609	0.307	0.624 / 0.911	0.84 / 0.208	0.16 / 0.792
TAPE Model 4	0.6296	0.234	0.599 / 0.925	0.871 / 0.226	0.129 / 0.774
One-hot Model 4	0.5988	0.143	0.547 / 0.907	0.742 / 0.116	0.258 / 0.884
TAPE Model 5	0.8344	0.482	0.727 / 0.948	0.947 / 0.457	0.053 / 0.543
One-hot Model 5	0.7937	0.451	0.701 / 0.923	0.902 / 0.297	0.098 / 0.703

Accuracy, precision and FDR were measured at positive : negative = 1:1 / 1:20

h

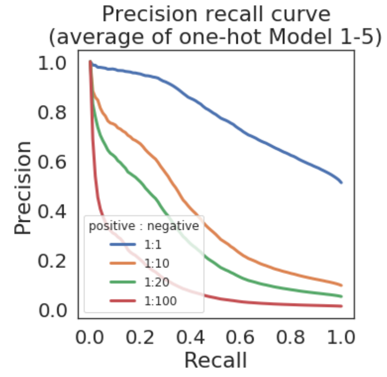

Fig. S1

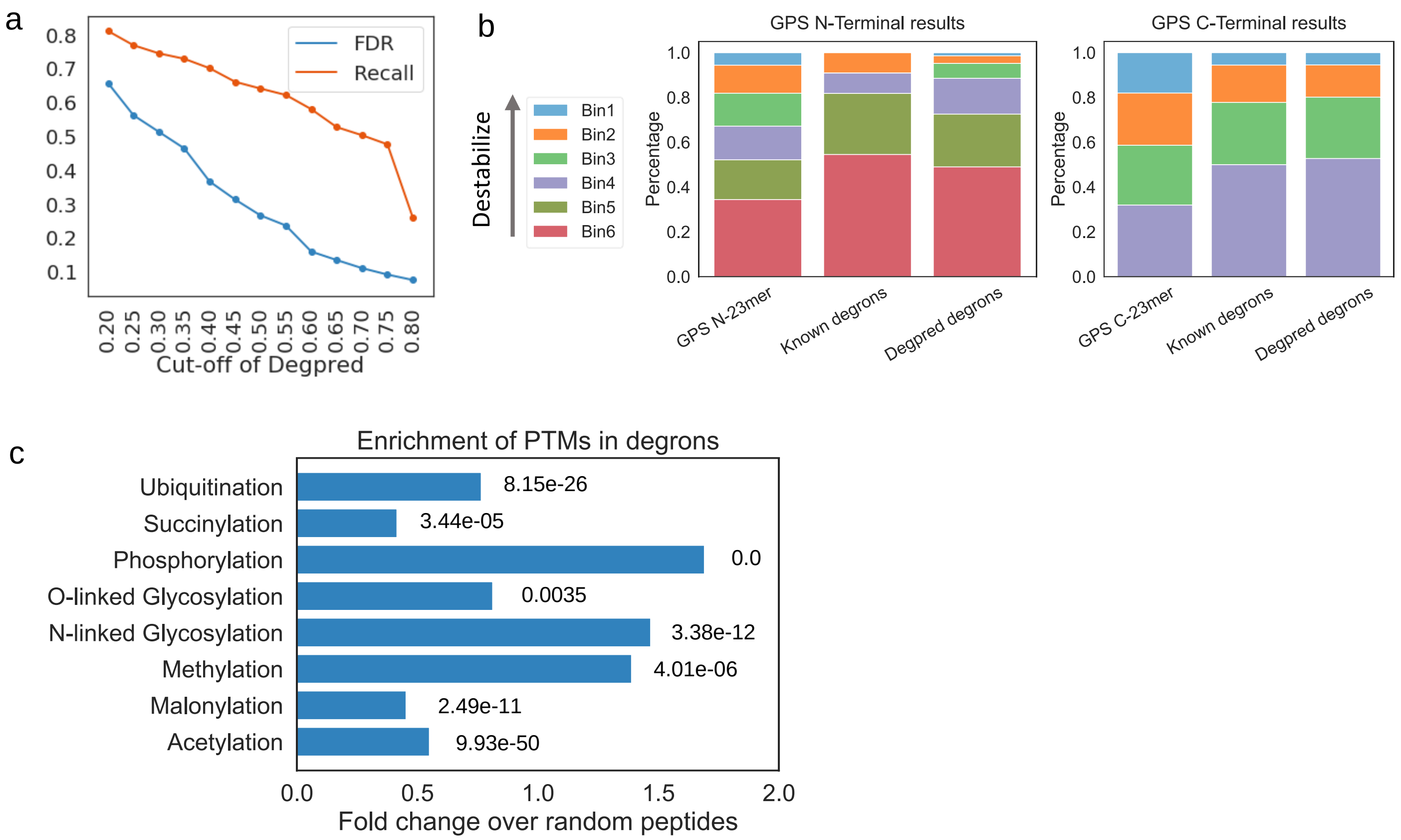

a
FDR
Recall
0.8
0.7
0.6
0.5
0.4
0.3
0.2
0.1
0.20 0.25 0.30 0.35 0.40 0.45 0.50 0.55 0.60 0.65 0.70 0.75 0.80
Cut-off of Degpred
b
Destabilize
Bin1
Bin2
Bin3
Bin4
Bin5
Bin6
GPS N-Terminal results
GPS C-Terminal results
Percentage
1.0
0.8
0.6
0.4
0.2
0.0
GPS N-23mer
Known degrons
Degpred degrons
GPS C-23mer
c
Enrichment of PTMs in degrons
Ubiquitination
Succinylation
Phosphorylation
O-linked Glycosylation
N-linked Glycosylation
Methylation
Malonylation
Acetylation
8.15e-26
3.44e-05
0.0
0.0035
3.38e-12
4.01e-06
2.49e-11
9.93e-50
0.0 0.5 1.0 1.5 2.0
Fold change over random peptides

Fig. S2

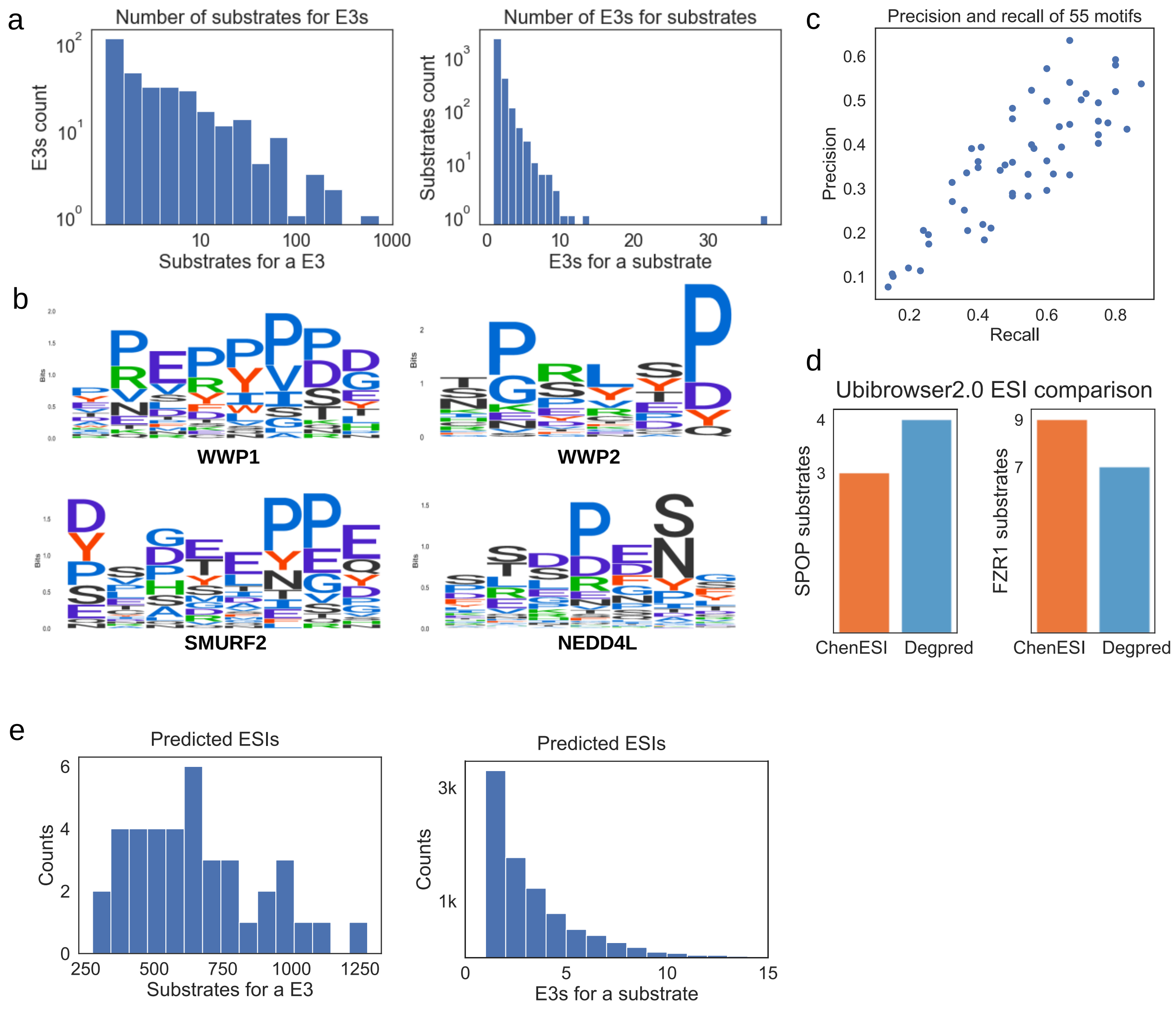

a
Number of substrates for E3s
E3s count
Substrates for a E3
Number of E3s for substrates
Substrates count
E3s for a substrate
b
WWP1
WWP2
SMURF2
NEDD4L
c
Precision and recall of 55 motifs
Precision
Recall
d
Ubibrowser2.0 ESI comparison
SPOP substrates
FZR1 substrates
ChenESI
Degpred
e
Predicted ESIs
Counts
Substrates for a E3
E3s for a substrate

Fig. S3

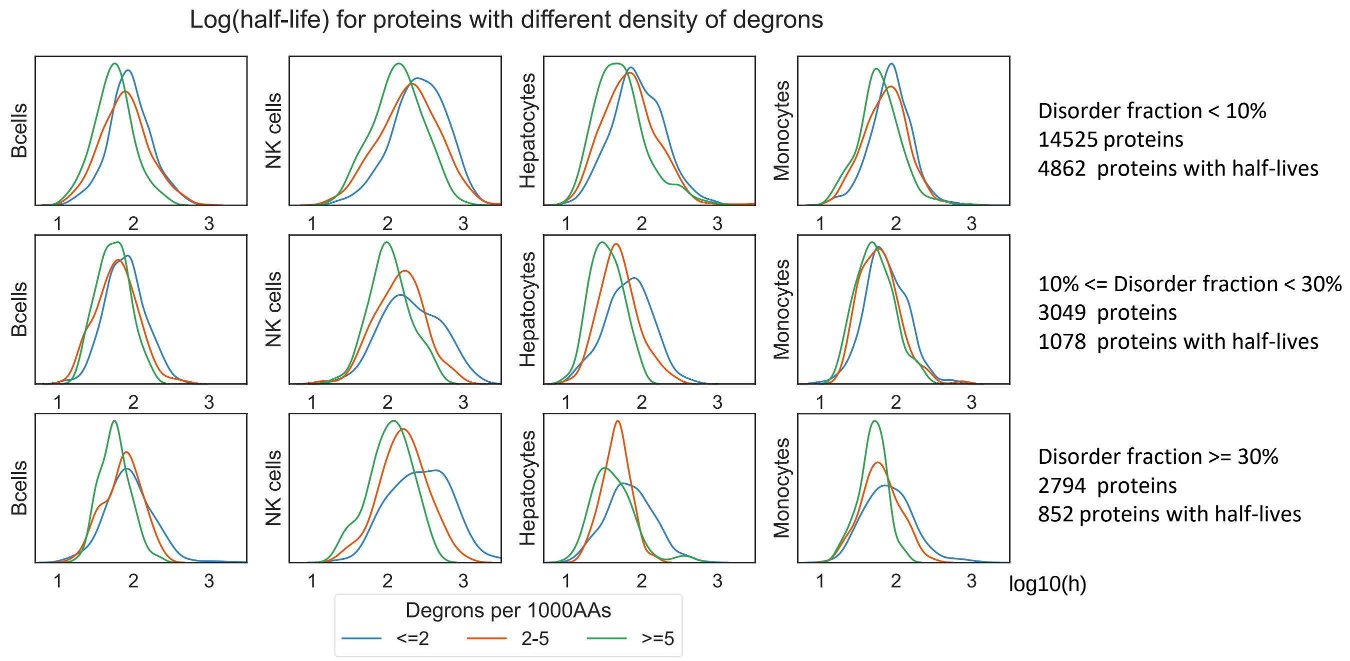

Log(half-life) for proteins with different density of degrons
Bcells
NK cells
Hepatocytes
Monocytes
1
2
3
Disorder fraction < 10%
14525 proteins
4862 proteins with half-lives
10% <= Disorder fraction < 30%
3049 proteins
1078 proteins with half-lives
Disorder fraction >= 30%
2794 proteins
852 proteins with half-lives
log10(h)
Degrons per 1000AAs
<=2
2-5
>=5

Fig. S4

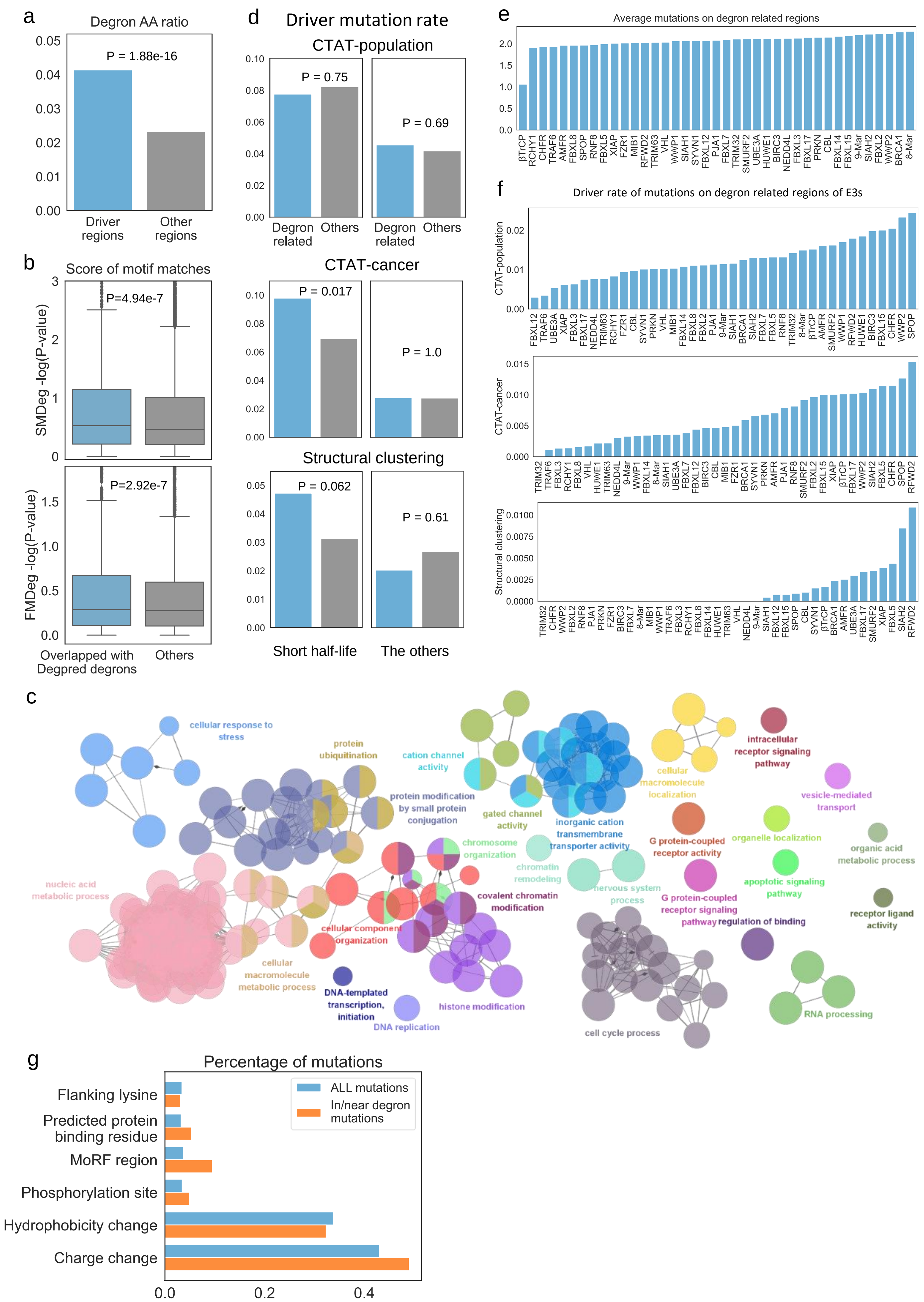
a
Degron AA ratio
P = 1.88e-16
Driver regions
Other regions
b
Score of motif matches
P=4.94e-7
SMDeg -log(P-value)
P=2.92e-7
FMDeg -log(P-value)
Overlapped with Degpred degrons
Others
d
Driver mutation rate
CTAT-population
P = 0.75
P = 0.69
Degron related
Others
CTAT-cancer
P = 0.017
P = 1.0
Structural clustering
P = 0.062
P = 0.61
Short half-life
The others
e
Average mutations on degron related regions
f
Driver rate of mutations on degron related regions of E3s
CTAT-population
CTAT-cancer
Structural clustering
c
cellular response to stress
protein ubiquitination
cation channel activity
protein modification by small protein conjugation
gated channel activity
inorganic cation transmembrane transporter activity
cellular macromolecule localization
intracellular receptor signaling pathway
vesicle-mediated transport
organelle localization
organic acid metabolic process
G protein-coupled receptor activity
chromosome organization
chromatin remodeling
nervous system process
G protein-coupled receptor signaling pathway
apoptotic signaling pathway
regulation of binding
receptor ligand activity
nucleic acid metabolic process
covalent chromatin modification
cellular component organization
cellular macromolecule metabolic process
DNA-templated transcription, initiation
DNA replication
histone modification
cell cycle process
RNA processing
g
Percentage of mutations
ALL mutations
In/near degron mutations
Flanking lysine
Predicted protein binding residue
MoRF region
Phosphorylation site
Hydrophobicity change
Charge change

Fig. S5
