# Supplementary material for: Systematic prediction of degrons and E3 ubiquitin ligase binding via deep learning
Source: BMC Biol. 2022 Jul 14;20:162. doi: 10.1186/s12915-022-01364-6 (PMC9281121; doi:10.1186/s12915-022-01364-6)
Supplement: Supplementary file 8 — Additional file 8. Uncropped western blots. [file 12915_2022_1364_MOESM8_ESM.pdf]

Systematic Prediction of Degrons and E3 Ubiquitin Ligase Binding via Deep Learning

This file contains uncropped western blots

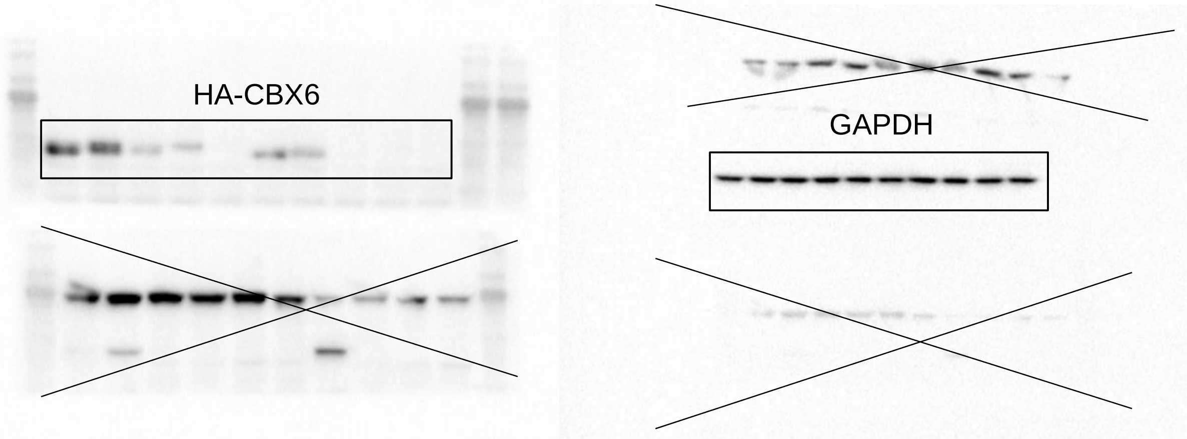

Uncropped western blot images of Fig. 4c

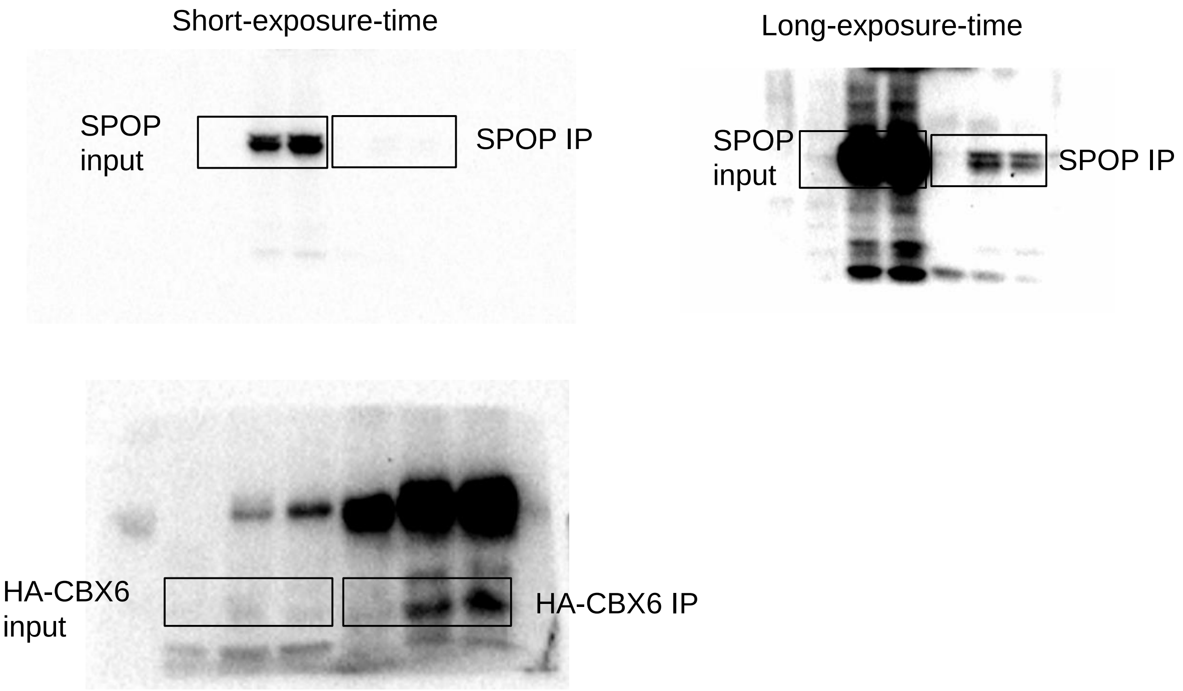

Uncropped Western blot images of Fig. 4d

It should be noted that for SPOP bands, we put short-exposure-time version for input bands and long-exposure-time version for IP bands since input bands are overexposed after a longer exposure time. This will not affect the conclusions derived from our results.
